# One-year oral toxicity study on a genetically modified maize MON810 variety in Wistar Han RCC rats (EU 7th Framework Programme project GRACE)

**DOI:** 10.1007/s00204-016-1798-4

**Published:** 2016-07-20

**Authors:** Dagmar Zeljenková, Radka Aláčová, Júlia Ondrejková, Katarína Ambrušová, Mária Bartušová, Anton Kebis, Jevgenij Kovrižnych, Eva Rollerová, Elena Szabová, Soňa Wimmerová, Martin Černák, Zora Krivošíková, Miroslava Kuricová, Aurélia Líšková, Viera Spustová, Jana Tulinská, Mikuláš Levkut, Viera Révajová, Zuzana Ševčíková, Kerstin Schmidt, Jörg Schmidtke, Paul Schmidt, Jose Luis La Paz, Maria Corujo, Maria Pla, Gijs A. Kleter, Esther J. Kok, Jutta Sharbati, Marc Bohmer, Nils Bohmer, Ralf Einspanier, Karine Adel-Patient, Armin Spök, Annette Pöting, Christian Kohl, Ralf Wilhelm, Joachim Schiemann, Pablo Steinberg

**Affiliations:** 1 Faculty of Public Health, Slovak Medical University in Bratislava, Limbová 12, 83303 Bratislava, Slovakia; 2 Faculty of Medicine, Slovak Medical University in Bratislava, Limbová 12, 83303 Bratislava, Slovakia; 3 University of Veterinary Medicine and Pharmacy in Košice and TOPALAB, Kamenicna 7, 01015 Košice, Slovakia; 4 BioMath GmbH, Schnickmannstr. 4, 18055 Rostock, Germany; 5 Centre for Research in Agricultural Genomics (CRAG), Edifici CRAG, Campus UAB, 08193 Cerdanyola, Barcelona Spain; 6 Universitat de Girona (UDG), Edifici EPS1, Campus Montilivi, 17071 Girona, Spain; 7 RIKILT Wageningen UR, Wageningen University and Research Centre, Akkermaalsbos 2, 6708WB Wageningen, The Netherlands; 8 Institute of Veterinary Biochemistry, Freie Universität Berlin, Oertzenweg 19b, 14163 Berlin, Germany; 9 INRA, UR496 Immuno-Allergie Alimentaire, CEA/IBiTeC-S/SPI, CEA de Saclay, 91191 Gif Sur Yvette Cedex, France; 10 IFZ-Inter-University Research Centre for Technology, Work and Culture (IFZ), Schlögelgasse 2, 8010 Graz, Austria; 11 Federal Institute for Risk Assessment, Max-Dohrn-Straße 8-10, 10589 Berlin, Germany; 12 Institute for Biosafety in Plant Biotechnology, Julius Kühn-Institut, Federal Research Centre for Cultivated Plants, Erwin-Baur-Str. 27, 06484 Quedlinburg, Germany; 13 Institute for Food Toxicology and Analytical Chemistry, University of Veterinary Medicine Hannover, Bischofsholer Damm 15, 30173 Hannover, Germany

**Keywords:** Food/Feed Guidance Document of the EFSA Scientific Committee (2011), Genetically modified maize MON810, GRACE, OECD Test Guideline No. 452–Chronic toxicity studies (2009), Rat feeding trial, Chronic oral toxicity study

## Abstract

The GRACE (GMO Risk Assessment and Communication of Evidence; www.grace-fp7.eu) project was funded by the European Commission within the 7th Framework Programme. A key objective of GRACE was to conduct 90-day animal feeding trials, animal studies with an extended time frame as well as analytical, in vitro and in silico studies on genetically modified (GM) maize in order to comparatively evaluate their use in GM plant risk assessment. In the present study, the results of a 1-year feeding trial with a GM maize MON810 variety, its near-isogenic non-GM comparator and an additional conventional maize variety are presented. The feeding trials were performed by taking into account the guidance for such studies published by the EFSA Scientific Committee in 2011 and the OECD Test Guideline 452. The results obtained show that the MON810 maize at a level of up to 33 % in the diet did not induce adverse effects in male and female Wistar Han RCC rats after a chronic exposure.

**Electronic supplementary material:**

The online version of this article (doi:10.1007/s00204-016-1798-4) contains supplementary material, which is available to authorized users.

## Introduction

The GRACE (GMO Risk Assessment and Communication of Evidence; www.grace-fp7.eu) project is a collaborative project, which involved 19 partners from 13 different European countries and was funded by the European Commission within the 7th Framework Programme between July 2012 and November 2015. A key objective of GRACE was to test GM maize MON810 varieties in subchronic and chronic animal feeding trials and alternative in vitro methods in order to determine how suitable the above-mentioned test systems are and whether they provide useful scientific information for the health risk assessment of GM food and feed in an “untargeted approach”, i.e. without a triggering indication of potential effects.

In the present study, the results of a 1-year feeding trial with a GM maize MON810 variety, its near-isogenic non-GM comparator and an additional conventional maize variety are presented. The transgenic trait MON810 consists of a *Bacillus thuringiensis* (*Bt*)-derived gene, namely a truncated *cry1Ab* gene encoding an insecticidal protein (δ-endotoxin) (Schnepf et al. 1998), for the control of some lepidopteran insect pests such as the European corn borer (*Ostrinia nubilalis*) (Hill et al. 1995). The selected MON810 variety was one of the most widely used by farmers in Catalonia, Spain. In the 1-year feeding trial, not only the corresponding near-isogenic non-GM maize comparator but also a conventional maize variety was tested, since the Slovak Medical University (Bratislava, Slovakia), the institution conducting the feeding trials, had not performed such an extended study with maize in the past and, therefore, appropriate historical data with which one could compare the results obtained in the present study were lacking.

The study design was derived from the OECD Test Guideline 452 for Testing of Chemicals–Chronic toxicity studies (OECD TG 452; OECD 2009) and took into account recommendations of the European Food Safety Authority (EFSA) when wanting to analyse the potential subchronic toxicity of whole food and feed in rodents (EFSA Scientific Committee 2011). The composition of the feed was analysed, the body weight and the feed consumption were monitored, clinical and ophthalmological observations were recorded, haematology and clinical biochemistry parameters were quantified, and a gross necropsy including the determination of the absolute and relative organ weights and a histopathological analysis were performed.

## Materials and methods

### Plant material

Maize was produced in Pla de Foixà (Girona, Catalonia, Spain, 42°05′N, 3°E) during the growing season of 2013. This area is close to the sea and has a Mediterranean climate. The soil type is Xerofluven oxiaquic, coarse-loamy, mixed, calcareous and thermic. Three varieties were produced, all commercially cultivated in that region: a GM maize MON810 and its near-isogenic non-GM comparator as well as an additional conventional variety (Table 1). The seeds were purchased at the local market. About 1.5 ha of each variety was sown. There was no maize cultured in neighbouring fields that had been sown at the same period of time, so that the probability of cross-pollination was minimized. Maize was cultivated following standard agricultural practices in the area, including N, P and K fertilization (up to 300, 100 and 175 kg/ha, respectively). Weeds were controlled by pre-emergence application of 4 L HARNESS^®^ GTZ per ha (41 % Acetochlor + 19.5 % Terbuthylazine) and by post-emergence application of 0.6 L ELITE PLUS per ha (4 % nicosulfuron) and 1 L Callisto^®^ per ha (480 g/L mesotrione) or MUSTANG (30 % 2,4-D and 0.6 % florasulam), if needed. No insecticide was applied. In-furrow irrigations were supplied when needed during the cropping season. Maize was planted at a density of 80,000 plants ha^−1^ with 75-cm row spacing and 14-cm seed spacing.

**Table 1:** Maize variety content of the different diets used in the 1-year rat feeding trial

Diet	Maize variety content (%)
33 % near-isogenic non-GM maize	33 % DKC6666^a^
11 % MON810	11 % DKC6667-YG^b^ + 22 % DKC6666
33 % MON810	33 % DKC6667-YG
33 % conventional 2	33 % SY NEPAL^c^

^a^Near-isogenic maize variety of DKC6667-YG, from Monsanto

^b^Transgenic maize variety (MON810), from Monsanto

^c^Conventional maize variety, from Koipesol Semillas

Agronomic, morphologic, phenological and health parameters were monitored and were as usual in the region. Specifically, *Sesamia nonagrioides* and *Ostrinia nubilalis* infestations were not detected in the near-isogenic (DKC6666) and the GM (DKC6667-YG) maize, but 3.3 % of SY-NEPAL plants were infested. There was some fungal infection in non-GM plants, and *Fusarium* spp. was detected in 2.5 and 1.3 % of DKC6666 and SY-NEPAL stalks, respectively. There was no relevant viral infection. A good yield (i.e. 12,000–13,000 kg/ha) was achieved. Meteorological data were recorded (Electronic Supplementary Material, Fig. 1). The central part of each plot was independently harvested, and kernels were removed from the cobs on-site by machine. They had grain moisture levels in the usual range and were dried in a biological dryer, kept below 60 °C, down to a moisture level of 13–14 %.

**Fig. 1:**
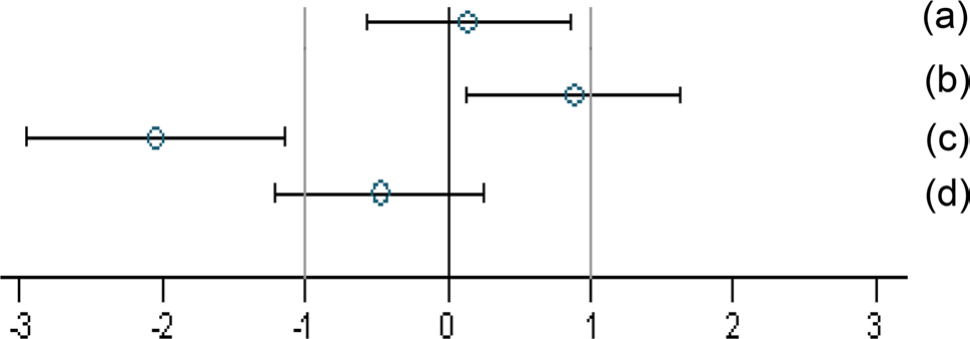
Simplified version of a graph allowing visual assessment of statistical significance as well as the supposed biological and possible toxicological relevance of group comparisons. The standard effect size point estimate (*circle*) and the 95 % confidence limits (*whiskers*, *bars* show confidence interval) illustrate the (standardized) effect size between two groups. The *vertical black line* indicates no effect (zero difference), while the *vertical grey lines* indicate the supposed biological and possible toxicological relevance limits (here ±1.0 SD, according to the study design). If the confidence *interval bars* cross the zero line but not the *grey lines* (lie within the ±1.0 limits), there is evidence for no statistical significance as well as no biological relevance (case *a*). Two groups are significantly different when the confidence interval bars do not cross the *black vertical line* (cases *b*, *c*). The effect size between two groups is supposed to be potentially relevant, when the confidence interval bars lie outside the ±1.0 SD limits (case *c*). Case *b* indicates statistical significance, but no clear biological relevance. Case *d* indicates no statistical significance, but no clear negation of biological relevance. This figure is Fig. 1 of the study by Zeljenková et al. (2014)

### Diet preparation and analyses

Two tons of DKC6666 maize, two tons of DKC6667-YG maize and one ton of SY-NEPAL maize were transported to Mucedola srl (Milan, Italy). The kernels were then milled (mesh size: 1 mm), coded and used to prepare the feed. The formulation of the diets was standard for all trials of the project (Zeljenková et al. 2014) except for the maize varieties used. It was isoproteic, isocaloric and adjusted to the dietary requirements of the rat strain Wistar Han RCC used in the feeding trials. Besides the milled maize, the formulation mainly consisted of other plant-derived ingredients, including wheat, wheat middlings, soybean meal and soy oil, while it did not contain animal-derived ingredients. Four different diets in pellet form (Table 1) were prepared in two batches, whereby the resulting pellets were dried at a temperature <50 °C, coded in a blinded fashion and sent to the Slovak Medical University (Bratislava, Slovakia) for the feeding trials as vacuum-packed, γ-irradiated batches (irradiation dose = 25 kGy).

Both batches of all four diets were analysed. Before dispatched to RIKILT Wageningen UR (Wageningen University and Research Centre, Wageningen, The Netherlands), small maize and diet subsamples were retained at the animal feed producing facility (Mucedola srl) for analysis. Diet samples of 1.5 kg each were sent to RIKILT Wageningen UR, where the feed pellets were milled and re-mixed. Thereafter, subsamples were dispatched to Covance (Madison, WI, USA). A list of the parameters measured, the analytical methods used and the institutions that performed the individual analyses is shown in Table 1 of the Electronic Supplementary Material. The feed analyses were performed in certified laboratories.

### Study design

The sample size of 20 rats per group, as described in the OECD TG 452 (OECD 2009), was chosen. A power analysis revealed that this group size would have a 85 % chance of detecting a standardized effect size (SES: the difference in means between control and treated groups divided by the standard deviation [SD]) of 1.0 SD by assuming the cage with two rats to be the experimental unit, at a 5 % significance level and by performing a two-sided test to compare the effects of the control and GMO diets in rats.

The total number of animals was 160, with 20 animals (10 cages) per gender and dietary treatment. Three dietary treatments represent the groups “control”, “11 % GMO” and “33 % GMO”. An additional group being fed a conventional maize variety with the same sample size per gender and group was included. Consequently, the factor “group” has four levels, namely “control”, “11 % GMO”, “33 % GMO” and “conventional 2”.^1^

### Experimental unit

As recommended by EFSA (EFSA Scientific Committee 2011), two animals of the same gender were housed per cage and the cage was taken as the experimental unit.

### Rat feeding trial

The rat feeding trial was conducted by taking into account the OECD TG 452 (OECD 2009) and recommendations included in the EFSA Guidance on conducting repeated-dose 90-day oral toxicity study in rodents on whole food/feed (EFSA Scientific Committee 2011). The trials were performed in compliance with GLP in the experimental animal house at the Department of Toxicology of the Slovak Medical University in Bratislava (Slovakia). Five-week old male and female Wistar Han RCC rats were purchased from Harlan (San Pietro al Natisone, Italy), and the study was started 1 week after delivery of the animals at the animal testing facility (i.e. in January 2014). Twenty animals with a uniform weight (±3–5 % of the mean) per group were used, two animals were placed in one cage (=experimental unit), and each animal was allocated to the individual cages by dose group and sex in such a way that the average weight between the treatment groups was similar. The feeding trial was started as follows: (1) feeding start for males on day 1 and (2) feeding start for females 1 day later. A detailed examination of all animals to verify their health condition (see “Periodical health status observations” section) was carried out just before the start of the feeding trials. Feed and water were supplied ad libitum. Feed consumption was determined once weekly during the first 13 weeks, every 2 weeks thereafter, and reported as the total amount of feed consumed by two animals in one cage per week or 2 weeks, respectively.

As mentioned before, two batches of each individual diet were produced. In the case of the male rats, the 2nd batch of the 33 % GMO and conventional 2 diets was fed from week 34 onwards, while the 2nd batch of the control and 11 % GMO diets was fed from week 33 onwards. In the case of the female rats, the 2nd batch of the control and 33 % GMO diets was fed from week 34 onwards, while the 2nd batch of the 11 % GMO and conventional 2 diets was fed from week 33 onwards.

### Periodical health status observations

Rats were inspected twice daily for changes in skin, fur, eyes, mucous membranes, occurrence of secretions and excretions as well as activity level and change in behaviour. A detailed physical examination of each animal out of the cage was performed prior to the beginning of the feeding trial, on day 1, once weekly during the first 13 weeks and once monthly thereafter to identify changes in skin, fur, eyes, mucous membranes, occurrence of secretions and excretions, and autonomic activity such as lacrimation, piloerection, pupil size, unusual respiratory patterns as well as activity level and change in behaviour. At the end of the feeding trials, a functional assessment of changes in gait, posture and response to handling as well as the presence of clonic or tonic movements or bizarre behaviour (self-mutilation, walking backwards) was carried out. Sensory reactivity to auditory, visual and proprioceptive stimuli was recorded. An ophthalmologic examination of both eyes of all animals in the conscious state was performed prior to the beginning of the feeding trial and 2 weeks before the end of the study. The eyes and the peribulbar structures were examined macroscopically after pupillary dilatation induced by instillation of a 0.5 % tropicamide solution. Each animal was weighed 48 h after its arrival at the experimental animal house of the Slovak Medical University, on the randomization day (i.e. one day before the beginning of the feeding trial), on the first day of the feeding trial, once weekly during the first 13 weeks, once every 2 weeks thereafter and at the end of the study.

### Haematology and clinical biochemistry analyses

At the end of months 3 and 6, blood samples from the tail vein of 10 males and 10 females per group after 16–18 h fasting were taken for the haematological analyses (with EDTA as anticoagulant) as well as for the clinical biochemistry analyses (without anticoagulant), thereby making use of the same animals at both points in time. At month 12, samples were taken from all animals in the 4 groups after 16–18 h fasting for the haematological analyses (with EDTA as anticoagulant) as well as for the clinical biochemistry analyses (without anticoagulant).

The order in which blood samples were taken for the haematology analyses is shown in Table 2 of the Electronic Supplementary Material. No later than four hours after collection of the blood samples, the following haematology parameters were measured by making use of a Sysmex K-4500 automated haematology analyser (Sysmex, Kobe, Japan): white blood cell count (WBC), red blood cell count (RBC), haemoglobin concentration (HGB), haematocrit (HCT), mean cell volume (MCV), mean corpuscular haemoglobin (MCH), mean corpuscular haemoglobin concentration (MCHC), platelet count (PLT) and lymphocyte count (LYM). For the differential leucocyte count, blood smears were stained with the May–Grunwald and Giemsa–Romanowski dyes and thereafter examined by light microscopy; the percentage of lymphocytes, neutrophils, eosinophils, basophils and monocytes were determined by examining 200 cells.

**Table 2:** Cry1Ab levels the different diets used in the 1-year feeding trial

	Control	Conventional 2	11 % GMO	33 % GMO
First batch of diets				
MON810 maize event–genetically modified DNA (%)	Detected, not quantifiable	Detected, not quantifiable	10.4	31.3
Cry1Ab (ng/mg protein)	Not detected	Not detected	0.6	1.27
Second batch of diets				
MON810 maize event–genetically modified DNA (%)	Not detected	Not detected	12.8	40.2
Cry1Ab (ng/mg protein)	Not detected	Not detected	0.47	1.2

The order in which blood samples were taken for the biochemistry analyses is also shown in Table 2 of the Electronic Supplementary Material. The parameters alkaline phosphatase (ALP), alanine aminotransferase (ALT), aspartate aminotransferase (AST), albumin (ALB), total protein (TP), glucose (GLU), creatinine (CREA), urea (U), cholesterol (CHOL), triglycerides (TRG), calcium (Ca), chloride (Cl), potassium (K), sodium (Na) and phosphorus (P) were measured maximally 4 h after collection of the blood samples in serum with an Ortho Clinical Vitros^®^ 250 Chemistry System (Ortho-Clinical Diagnostics, Raritan, NJ, USA), whereas coagulation parameters were not determined.

### Urinalysis

An analysis of urine was performed at months 3, 6 and 12 on 10 male and 10 female rats using the same animals throughout. Urine was collected from each individual rat in metabolic cages for 16 h. The parameters total protein, glucose, ketone, leucocyte number, erythrocyte number, bilirubin, urobilinogen, nitrate and pH were analysed with Combur^10^Test^®^ UX test strips (Roche Diagnostics, Mannheim, Germany) and semi-quantitatively evaluated by reflectance photometry with a Urilux S analyser (Roche Diagnostics). Osmolality was measured with the Advanced^®^ Model 3300 micro-osmometer from Advanced Instruments (Norwood, MA, USA).

### Gross necropsy and histopathology

At the end of the study, rats were anaesthetized after a 16- to 18-h fasting period with 10 mg/kg bw xylazine and 75 mg/kg bw ketamine. The order in which necropsy was performed is also shown in Table 2 of the Electronic Supplementary Material. Blood samples were taken from the abdominal aorta and in four cases from the inferior vena cava for possible omics analyses being outside the scope of this publication. Thereafter, the successive necropsy of the thoracic cavity, the abdominal cavity, the genital organs and, following decapitation, the head was performed. Moreover, the wet weight of the kidneys, spleen, liver, adrenal glands, lung, heart, thymus, pancreas, uterus, ovaries, testes, epididymides and brain of all animals was recorded. Organ samples were stored in neutrally buffered 10 % formalin, except for the eyes and the male reproductive tissues, which were immersed in Bouin’s solution, and sent to TOPALAB (Košice, Slovakia) for their histopathological examination.

A complete microscopic examination of the brain (including cerebrum, cerebellum and medulla/pons), spinal cord (at the cervical, mid-thoracic and lumbar level), pituitary, thyroid, parathyroid, thymus, oesophagus, salivary glands, stomach, small and large intestines, liver, pancreas, kidneys, adrenals, spleen, heart, trachea and lungs (preserved by inflation with fixative and then immersion), aorta, gonads, uterus, female mammary gland, prostate, urinary bladder, lymph nodes, peripheral nerve, bone marrow and skin from all animals in the control and high dose groups was performed. In order to do so, the formalin-fixed tissue samples were washed, dehydrated and embedded in paraffin. Thereafter, 4-µm-thick sections were stained with haematoxylin and eosin for the light microscopic examination of the tissue structure. All tissues from animals killed before the end of the study (animal Nos. 15, 131 and 159), and all tissues from animals fed the conventional 2 and 11 % GMO diets showing macroscopic alterations were also examined (see Electronic Supplementary Material Tables 8 and 9).

### Statistics

The raw data of the trial were collated in Excel files. Data were screened for their structure, and data and variable definitions were settled (Appendix 1 in Schmidt et al. 2015a). Based on these definitions, a SAS analysis data set was created by using SAS Software version 9.4 (SAS Institute Inc., Cary, NC). Mean values per cage were calculated for all endpoints except for feed consumption. The feed efficiency per week until week 13 and per 2 weeks thereafter (weight gain [g]/feed intake [g] × 100) and the relative organ weights (organ weight [g]/body weight [g] × 100) were computed. Data were screened for outliers and extreme values (Appendix 2 in Schmidt et al. 2015a). For each gender–group, factor level combination and all variables, box-and-whisker plots were created to identify extreme values (variable values outside 1.5* interquartile range). Extreme values were marked in the Excel sheet of original data for easier identification of irregular patterns or suspicious animals. Growth curves of all animals were plotted (scatter plots, weight against study day) and visually inspected for irregular patterns (Appendix 2 in Schmidt et al. 2015a). To describe the data, summary statistics including means, standard deviations, 95 % confidence intervals, medians, number of valid values, minima and maxima were calculated and tabulated. Additionally to the box-and-whisker plots, plots of means and 95 % confidence intervals were drawn. Descriptive analysis was performed separately for each gender and group and on an animal basis (Appendix 3 in Schmidt et al. 2015a).

The body weight, feed consumption and feed efficiency data (per cage) were analysed by applying mixed models and by using the restricted maximum likelihood (REML) algorithm with AR covariance structure, combined and separately for male and female animals. The group (four levels) was considered a fixed factor. The factor week (time in weeks from the start of the experiment) was considered a continuous fixed factor (Appendix 4 in Schmidt et al. 2015a). For least square mean body weights and for all other endpoints, SES and their 95 % confidence intervals were calculated for each observation date (i.e. 3, 6 and 12 months) according to Nakagawa and Cuthill (2007; for details, see Schmidt et al. 2015b). The GMO and the conventional 2 groups were compared to the control group (three comparisons: 11 % GMO–control, 33 % GMO–control and conventional 2–control) (Appendix 5 in Schmidt et al. 2015a). The SES estimates are displayed as graphs displaying both statistical significance and the ±1.0 SD interval (as assumed in the sample size calculation based on an EFSA guidance document [EFSA Scientific Committee 2011]) for each of the endpoint comparison results (Fig. 1). All endpoints are shown on the same graph (separately for the male and female rats), thereby forming an overall pattern and allowing the assessment of group comparisons at a glance. In addition, in order to compare the temporal courses of the haematology and clinical biochemistry endpoints (per cage), mixed models and the restricted maximum likelihood (REML) algorithm with AR covariance structure were applied. The group (four levels) was considered a fixed factor. The factor time (observation points in time at 3, 6 and 12 months) was considered a continuous fixed factor (Appendix 6 in Schmidt et al. 2015a).

A “classical” statistical analysis was also performed. In a first step, the generic assumptions underlying the ANOVA post hoc tests were checked: for the normality of the data, Kolmogorov–Smirnov (with Lilliefors correction) tests were performed. For residuals, scaled-by-predicted plots, residual histograms and residual QQ plots were generated (Appendix 7 in Schmidt et al. 2015a). For variance homogeneity, Levene’s test was performed. Based on this test and following a decision tree (OECD 2012; Schmidt et al. 2015b), the appropriate test procedure was chosen (Appendix 10 in Schmidt et al. 2015a): an ANOVA with post hoc Dunnett test was applied in the case of quantitative data being independent observations with normally distributed residuals and equal variances in the groups. For qualitative data and quantitative data, in which the ANOVA assumptions were not met, the Kruskal–Wallis followed by the Wilcoxon test was applied. Group means and standard deviations (data per cage) of all endpoints were listed in form of tables (Appendix 8 in Schmidt et al. 2015a). The significances obtained with the above-mentioned decision tree-based “classical” statistical analysis procedure as well as the significances identified by SES confidence intervals are shown in Tables 4, 5 and 8 listing the haematology, clinical biochemistry and relative organ weight data of the feeding trial, respectively.

To illustrate and compare the consequences of applying several parametric and nonparametric methods, all endpoints were analysed by three commonly used tests, namely an ANOVA followed by both the t and the Dunnett test as well as the Kruskal–Wallis test followed by the Wilcoxon test. The resulting significances were compared to each other and to the significances identified by SES confidence intervals (Appendices 9 and 10 in Schmidt et al. 2015a).

In this paper, when comparing haematology and clinical biochemistry parameters as well as relative organ weights between a control and a second group, the wording “significantly different” is based on the interpretation of the calculated SES estimates (Fig. 1). Furthermore, in those cases, in which the classical statistical analysis methods revealed differences between the control group and the 11 % GMO and/or 33 % GMO group not identified by the SES confidence intervals, these are mentioned in “Results” section.

### Stakeholder consultations

A key characteristic of the GRACE project is to allow for a broad involvement of stakeholders and to ensure utmost transparency of the research process. The main stakeholder groups targeted were competent authorities, industry, civil society organizations, and researchers interested or experienced in animal feeding studies with GM food/feed. The groups contacted were much broader and also included, for example, agricultural, professional and international organizations.

A draft study plan was sent per e-mail to 738 stakeholders. A total of 122 comments were received from eight individuals or organizations. These comments were discussed by the study team and taken into account when finalizing the study plan. Study team members answered in a written form the stakeholder comments, thereby allowing them to track if and how their comments were taken into consideration and to understand the underlying reasons for taking them into account or not.

In a similar way, the draft results, interpretations and conclusions were subjected to stakeholder scrutiny in the course of the final round of stakeholder consultations on GRACE results. More than 1300 stakeholders were contacted, 27 participated in a workshop held in October 2015, and 6 of the workshop participants provided additional 41 comments in writing. In order to facilitate this process, draft documents and raw data were made available to registered participants that had agreed to sign a non-disclosure agreement. Again, all comments were considered when completing interpretation and conclusions, and written responses were prepared by the study team.

All comments and the written responses of the study team members were documented in consultation reports and published at the project website alongside with the draft and revised study plan (http://www.grace-fp7.eu). Stakeholder participation was not selected in any way: all interested stakeholder representatives could participate. In each consultation step, representatives of all main stakeholder groups targeted were involved and actively contributing.

## Results

### Feed composition analysis

A detailed quantitative analysis of the different components of the diets used in the feeding trial was performed; the results of the analysis of the 1st batch are listed in Table 3 of the Electronic Supplementary Material and the results of the analysis of the 2nd batch in Table 4 of the Electronic Supplementary Material.

**Table 3:** Clinical observations in male and female Wistar Han RCC rats during the 1-year feeding trial

Group	Animal no.	Week no.	Clinical observations
Male rats			
33 % GMO	2	10	Diarrhoea
	7	25	Bite wounds on the left scapula and left ventral side
	15^a^	31	Paraplegia of the hind limbs
Control	50	6	Diarrhoea
Conventional 2	63	9	Diffuse epistaxis, chromodacryorrhea
	79	40	Exophthalmos (both eyes), diarrhea
Female rats			
33 % GMO	102	43	Chromodacryorrhea
	106	25	Hair loss between the scapulae
	107	23–53	Bite wound, pruritus and scabs between the scapulae
	109	26	Tail wound
11 % GMO	126	30	Bite wound between the scapulae
	129	42–53	Hair loss on the left side of the face
	131^a^	33–34	Weight loss, apathy, piloerection, hypersalivation, haematemesis, chromodacryorrhea, enlarged abdominal cavity, abdominalgia, dyspnoea
	135	26	Tail wound
Control	144	42–53	Maxilla deformation, exophthalmos, chromodacryorrhea, dyspnoea
	147	9	Bite wound on the back
	159^a^	31–47	Mammary gland abscess

^a^Rats 15, 131 and 159 were killed on days 227, 244 and 327, respectively

**Table 4:** Mean values ± standard deviations (SD) of haematology parameters in male and female Wistar Han RCC rats at 3, 6 and 12 months

Parameter	Males				Females			
	Control^1^	Conv. 2^2^	11 % GMO^3^	33 % GMO^4^	Control^5^	Conv. 2^6^	11 % GMO^7^	33 % GMO^8^
At 3 months								
WBC (10^3^/μl)	7.16 ± 0.83	6.44 ± 0.94	8.24 ± 1.89	7.71 ± 1.39	5.16 ± 1.45	6.65 ± 1.23	5.65 ± 1.58	6.71 ± 1.39
RBC (10^6^/μl)	8.12 ± 0.22	8.26 ± 0.34	7.98 ± 0.51	8.14 ± 0.39	7.71 ± 0.24	8.07 ± 0.25	7.27 ± 0.43	7.41 ± 0.43
HGB (g/dl)	16.09 ± 0.34	15.94 ± 0.72	16.05 ± 0.62	16.12 ± 0.74	15.15 ± 0.93	15.64 ± 0.38	14.83 ± 0.97	14.99 ± 0.81
HCT (%)	44.90 ± 1.17	45.11 ± 2.40	43.87 ± 2.85	45.25 ± 2.37	43.72 ± 1.46	45.04 ± 1.09	41.69 ± 2.40	41.71 ± 1.97
MCV (fl)	55.32 ± 0.89	54.64 ± 1.47	54.91 ± 0.65	55.68 ± 1.03	56.74 ± 1.16	55.84 ± 1.28	57.39 ± 0.39	56.34 ± 0.95
MCH (pg)	19.86 ± 0.44	19.32 ± 0.41	20.17 ± 1.07	19.85 ± 0.36	18.20 ± 4.24	19.39 ± 0.56	20.42 ± 0.75	20.26 ± 0.27
MCHC (g/dl)	35.94 ± 0.48	35.35 ± 0.39	36.79 ± 2.30	35.64 ± 0.43	31.93 ± 7.30	34.73 ± 0.51	35.59 ± 1.12	35.95 ± 0.34
PLT (10^3^/μl)	678.00 ± 140.75	717.70 ± 34.10	766.90 ± 112.28	748.60 ± 143.33	699.40 ± 203.54	723.70 ± 99.60	656.50 ± 110.51	655.70 ± 114.11
LYM (10^3^/µl)	4.93 ± 0.82	4.42 ± 0.68	5.87 ± 1.33	5.95 ± 1.30	3.31 ± 0.84	4.40 ± 0.78	3.76 ± 1.01	4.42 ± 1.04
Lymphocytes (%)	71.00 ± 4.51	68.10 ± 5.39	70.80 ± 4.83	73.70 ± 3.51	70.45 ± 4.01	67.65 ± 4.79	69.85 ± 3.26	69.35 ± 6.89
Neutrophils (%)	23.90 ± 4.21	26.00 ± 3.81	23.85 ± 5.36	21.70 ± 2.72	25.00 ± 4.05	26.55 ± 4.02	24.60 ± 2.58	24.60 ± 6.09
Monocytes (%)	2.75 ± 0.64	4.10 ± 3.16	2.25 ± 0.59	3.05 ± 1.02	2.65 ± 0.63	3.20 ± 0.45	2.75 ± 0.61	2.35 ± 0.84
Eosinophils (%)	2.35 ± 0.89	1.80 ± 0.97	3.10 ± 1.59	1.55 ± 0.48	1.85 ± 0.52	2.55 ± 1.14	2.80 ± 0.93	3.65 ± 0.96^a,c^
Basophils (%)	0.00 ± 0.00	0.00 ± 0.00	0.00 ± 0.00	0.00 ± 0.00	0.05 ± 0.11	0.05 ± 0.11	0.00 ± 0.00	0.05 ± 0.11
At 6 months								
WBC (10^3^/μl)	7.06 ± 0.57	8.44 ± 2.09	8.48 ± 0.63^b,c^	9.10 ± 1.49^c^	6.06 ± 1.36	7.85 ± 0.87^b^	7.05 ± 1.44	7.02 ± 1.00
RBC (10^6^/μl)	8.34 ± 0.18	8.28 ± 0.16	8.07 ± 0.20	8.21 ± 0.25	7.69 ± 0.23	7.72 ± 0.42	7.36 ± 0.25	7.56 ± 0.44
HGB (g/dl)	16.13 ± 0.54	15.83 ± 0.46	15.39 ± 0.55	15.94 ± 0.52	15.55 ± 0.27	15.29 ± 0.50	15.23 ± 0.37	15.40 ± 0.65
HCT (%)	46.29 ± 1.29	45.62 ± 1.40	44.49 ± 1.41	45.48 ± 1.30	44.85 ± 0.95	44.16 ± 1.67	43.23 ± 1.10	43.69 ± 1.93
MCV (fl)	55.53 ± 1.26	55.10 ± 1.31	55.11 ± 0.57	55.47 ± 0.79	58.37 ± 1.14	57.27 ± 1.58	58.78 ± 0.70	57.81 ± 1.26
MCH (pg)	19.36 ± 0.59	19.12 ± 0.51	19.06 ± 0.37	19.47 ± 0.32	20.25 ± 0.66	19.84 ± 0.90	20.71 ± 0.31	20.42 ± 0.51
MCHC (g/dl)	34.85 ± 0.37	34.71 ± 0.51	34.60 ± 0.44	35.08 ± 0.33	34.69 ± 0.53	34.63 ± 0.71	35.24 ± 0.40	35.31 ± 0.36
PLT (10^3^/μl)	749.10 ± 52.24	785.90 ± 139.31	782.60 ± 147.63	629.80 ± 112.45	730.40 ± 78.00	798.60 ± 47.15	733.40 ± 75.11	732.00 ± 110.25
LYM (10^3^/µl)	5.55 ± 1.06	5.95 ± 1.70	6.74 ± 1.23	7.10 ± 1.89	3.87 ± 0.63	4.67 ± 0.78	4.65 ± 0.90	4.85 ± 0.69
Lymphocytes (%)	69.35 ± 4.16	63.60 ± 5.54	64.65 ± 4.70	65.20 ± 4.28	71.45 ± 5.90	64.25 ± 6.56	72.15 ± 4.95	77.85 ± 2.78
Neutrophils (%)	25.40 ± 3.99	31.85 ± 6.57	29.25 ± 4.27	30.45 ± 3.64	24.45 ± 5.83	30.55 ± 6.47	23.35 ± 5.01	18.95 ± 2.87
Monocytes (%)	2.75 ± 0.98	3.05 ± 0.82	4.00 ± 0.94	2.45 ± 0.65	1.90 ± 0.60	2.50 ± 0.64	2.45 ± 0.54	2.00 ± 0.61
Eosinophils (%)	2.45 ± 0.86	1.50 ± 0.83	2.10 ± 0.22	1.90 ± 0.72	2.20 ± 0.27	2.70 ± 0.80	2.05 ± 0.65	1.20 ± 0.57^b,c^
Basophils (%)	0.05 ± 0.11	0.00 ± 0.00	0.00 ± 0.00	0.00 ± 0.00	0.00 ± 0.00	0.00 ± 0.00	0.00 ± 0.00	0.00 ± 0.00

Parameter	Males				Females				
	Control^1^	Conv. 2^2^	11 % GMO^3^	33 % GMO^4^	Control^5^	Conv. 2^6^	11 % GMO^7^	33 % GMO^8^	
At 12 months									
WBC (10^3^/μl)	9.10 ± 1.18	8.06 ± 1.32	9.41 ± 2.26	8.88 ± 1.32	6.76 ± 1.50	7.02 ± 2.29	6.15 ± 1.54		6.73 ± 1.25
RBC (10^6^/μl)	8.41 ± 0.26	8.37 ± 0.21	8.30 ± 0.33	8.33 ± 0.51	7.55 ± 0.39	7.69 ± 0.37	7.43 ± 0.22		7.54 ± 0.19
HGB (g/dl)	16.59 ± 0.46	16.34 ± 0.92	16.42 ± 0.55	16.43 ± 0.33	15.93 ± 0.39	15.91 ± 0.39	15.90 ± 0.30		15.98 ± 0.45
HCT (%)	46.99 ± 1.49	46.82 ± 1.40	45.87 ± 1.63	45.71 ± 2.33	43.62 ± 2.11	44.01 ± 1.26	43.39 ± 1.12		43.59 ± 1.07
MCV (fl)	55.89 ± 1.32	55.99 ± 1.50	55.32 ± 1.04	55.01 ± 1.78	57.79 ± 0.94	57.33 ± 1.86	58.46 ± 0.79		57.80 ± 0.97
MCH (pg)	19.74 ± 0.64	19.54 ± 0.95	19.82 ± 0.71	19.85 ± 1.48	21.17 ± 1.07	20.75 ± 0.86	21.43 ± 0.51		21.18 ± 0.39
MCHC (g/dl)	35.30 ± 0.54	34.93 ± 2.14	35.84 ± 0.71	36.08 ± 2.48	36.62 ± 1.83	36.18 ± 0.54	36.66 ± 0.66		36.67 ± 0.39
PLT (10^3^/μl)	715.80 ± 139.47	760.30 ± 119.31	781.00 ± 124.22	746.10 ± 105.94	748.65 ± 107.91	792.40 ± 137.52	726.60 ± 133.61		773.25 ± 113.78
LYM (10^3^/µl)	6.03 ± 1.18	5.55 ± 1.43	6.68 ± 1.87	6.37 ± 0.82	4.43 ± 1.08	4.75 ± 1.45	4.23 ± 0.89		4.66 ± 0.81
Lymphocytes (%)	63.60 ± 4.73	63.80 ± 7.83	63.00 ± 3.46	64.88 ± 4.66	66.43 ± 6.12	64.40 ± 5.99	64.35 ± 5.94		65.60 ± 3.17
Neutrophils (%)	31.33 ± 4.42	30.13 ± 7.10	30.35 ± 3.44	29.20 ± 4.82	27.18 ± 6.64	31.08 ± 5.97	31.13 ± 5.08		29.30 ± 3.15
Monocytes (%)	3.48 ± 0.74	3.25 ± 0.59	3.98 ± 0.90	3.65 ± 0.75	3.20 ± 0.65	2.68 ± 1.39	2.88 ± 1.17		2.98 ± 1.04
Eosinophils (%)	1.60 ± 0.56	2.83 ± 1.50^a,c^	2.65 ± 0.49^b,c^	2.28 ± 1.02	3.20 ± 1.35	1.83 ± 1.09^a,c^	1.65 ± 0.92^a,c^		2.13 ± 1.03
Basophils (%)	0.00 ± 0.00	0.00 ± 0.00	0.03 ± 0.08	0.00 ± 0.00	0.00 ± 0.00	0.03 ± 0.08	0.00 ± 0.00		0.00 ± 0.00

*WBC* white blood cells, *RBC* red blood cells, *HGB* haemoglobin, *HCT* haematocrit, *MCV* mean cell volume, *MCH* mean corpuscular haemoglobin, *MCHC* mean corpuscular haemoglobin concentration, *PLT* platelets, *LYM* lymphocytes

^1^9, 10 and 20 (19 in the case of LYM) male rats fed the diet containing 33 % near-isogenic non-GM maize for 3, 6 or 12 months, respectively, were analysed

^2^10, 10 and 20 male rats fed the diet containing 33 % conventional 2 maize for 3, 6 or 12 months, respectively, were analysed

^3^10, 10 and 20 male rats fed the diet containing 11 % GMO maize for 3, 6 or 12 months, respectively, were analysed

^4^9, 10 and 19 male rats fed the diet containing 33 % GMO maize for 3, 6 or 12 months, respectively, were analysed

^5^10, 10 and 19 female rats fed the diet containing 33 % near-isogenic non-GM maize for 3, 6 or 12 months, respectively, were analysed

^6^10, 10 and 20 female rats fed the diet containing 33 % conventional 2 maize for 3, 6 or 12 months, respectively, were analysed

^7^10, 10 and 19 (18 in the case of PLT) female rats fed the diet containing 11 % GMO maize for 3, 6 or 12 months, respectively, were analysed

^8^10, 10 and 20 (18 in the case of PLT) female rats fed the diet containing 33 % GMO maize for 3, 6 or 12 months, respectively, were analysed

^a^Statistically significant difference to control group based on one-way ANOVA, post hoc *t* test (*p* < 0.05) and post hoc Dunnett test (*p* < 0.05)

^(a)^Statistically significant difference to control group (*p* < 0.05) based on one-way ANOVA and post hoc *t* test, post hoc Dunnett test not significant

^b^Statistically significant difference to control group (*p* < 0.05) based on Wilcoxon test

^c^Statistically significant difference to control group based on 95 % confidence interval of the SES

### Feed composition analysis of the 1st batch of the four diets

The 1st batch of all four diets showed similar levels of most of the analysed proximates (ash, total carbohydrates, fat, protein), starch, fibres, amino acids, fatty acids, minerals, vitamins, sugars, anti-nutrients and secondary metabolites. In the case of folic acid, the conventional 2 and 11 % GMO diets contained somewhat lower levels and the 33 % GMO diet a somewhat higher level if compared to the control diet. The lead level was somewhat higher in the 11 % GMO diet and somewhat lower in the 33 % GMO diet when compared to the control diet. The β-, γ- and δ-tocopherol levels were similar in the control and 11 % GMO diets, while they were lower in the conventional 2 and 33 % GMO diets. The glucose level was somewhat lower in the 11 % GMO and 33 % GMO diets, the maltose level lower in the conventional 2 and 11 % GMO diets, the raffinose as well as the sucrose levels lower in the conventional 2 and 11 % GMO diets and higher in the 33 % GMO diet and the stachyose level somewhat lower in the conventional 2 diet and somewhat higher in the 33 % GMO diet if compared to the control diet. The phytic acid level was similar in the control, 11 % GMO and 33 % GMO diets and higher in the conventional 2 diet. The trypsin inhibitor level was higher in the 33 % GMO diet than in the control diet. The levels of the soy isoflavones daidzin, genistin and glycitin were higher in the 33 % GMO diet, while those of genistein and glycitein were lower in the 33 % GMO diet when compared to those of the control diet. Very low amounts of atropine were detected in all four diets. None of the measured concentrations/detected differences were considered to affect the health of the rats in any way.

Low and similar amounts of polychlorinated dibenzo-*p*-dioxins and dibenzofurans, polychlorinated biphenyls, polycyclic aromatic hydrocarbons, most mycotoxins and nitrosamines were detected in the 1st batch of all four diets (Table 3, Electronic Supplementary Material). Among the mycotoxins analysed, fumonisins B_1_, B_2_ and B_3_ were present at clearly higher levels in the control diet than in the other three diets. Beauvericin was present in the control and 11 % GMO diets at levels slightly above the limit of quantitation, whereas deoxynivalenol was present in the control and conventional 2 diets slightly above the limit of quantitation. Residues of the pesticides deltamethrin, ethoxyquin, piperonyl butoxide and pirimiphos-methyl were detected in all diets, chlorpyrifos in the control diet and *N*-desethyl-pyrimiphos-methyl in the 33 % GMO diet, but at levels that were considered not to affect the health of the rats in any way (Table 3, Electronic Supplementary Material).

As expected, the MON810 event was detected in the 1st batch of diets containing 11 and 33 % of the GM MON810 maize at the DNA and the protein level (Table 2). Moreover, the 1st batch of the control and the conventional 2 diets contained non-quantifiable traces of the MON810 event (Table 2). The ratios for the MON810 event level and the Cry1Ab protein content between the diet containing 11 % GMO and the one containing 33 % GMO were 3 and 2.1, respectively (Table 2). It should be noted that the detection of the *cry1Ab* gene in the diet sample of the 33 % GM maize failed for an unknown reason, while the MON810 event was quantified and the protein itself was detected as expected. While it can only be speculated as to why the *cry1ab*-gene-specific PCR amplicon (described by Scholtens et al. 2013) could not be detected, hypothetical yet unverified contributing factors may include design (*e.g.* impaired binding of selected primer to primer target site) and suboptimal reaction conditions (temperature, master mix).

Following irradiation, microorganisms such as coliforms, *Enterobacteriaceae*, yeast and moulds were not detected in the diets (Table 3, Electronic Supplementary Material).

### Feed composition analysis of the 2nd batch of the four diets

The 2nd batch of the four diets showed similar levels of most of the analysed proximates (ash, total carbohydrates, fat, protein), starch, fibres, amino acids, fatty acids, minerals, vitamins, sugars, anti-nutrients and secondary metabolites. The 33 % GMO diet contained a higher level of folic acid if compared to the control diet. The arsenic and selenium levels were higher in the conventional 2, 11 % GMO and 33 % GMO diets when compared to the control diet, while the cadmium level was higher in the control diet than in the other three diets. The β-, γ- and δ-tocopherol levels were similar in the control and 11 % GMO diets, while they were lower in the conventional 2 and 33 % GMO diets. The δ-tocopherol level was higher in the control diet than in the other three diets. The maltose, raffinose, stachyose and sucrose levels were higher in the conventional 2, 11 % GMO and 33 % GMO diets when compared to the control diet. Trypsin inhibitor activity was measured in the conventional 2 and 11 % GMO diets. The levels of the soy isoflavones daidzein, daidzin, genistein and genistin were higher in the control diet than in the other three diets, while those of glycitin were similar in the conventional 2 and 11 % GMO diets and higher than in the control and 33 % GMO diets. Very low levels of atropine and ergot alkaloid concentrations slightly above the limit of quantification were detected in the control diet. None of the measured concentrations/detected differences were considered to affect the health of the rats in any way.

Low and similar amounts of polychlorinated dibenzo-*p*-dioxins and dibenzofurans, polychlorinated biphenyls, polycyclic aromatic hydrocarbons, most mycotoxins and nitrosamines were detected in the 2nd batch of all four diets (Table 4, Electronic Supplementary Material). Among the mycotoxins analysed, fumonisins B_1_, B_2_ and B_3_ were present at higher levels in the control diet than in the other three diets. Furthermore, beauvericin was present in the control and 11 % GMO diets, whereas deoxynivalenol was present in the four diets at levels slightly above the limit of quantitation. Residues of the pesticides deltamethrin, ethoxyquin, piperonyl butoxide and pirimiphos-methyl were detected in all diets, hexythiazox and *N*-desethyl-pirimiphos-methyl in the 33 % GMO diet and chlorpyrifos-methyl in the control diet, but at levels that were considered not to affect the health of the rats in any way (Table 4, Electronic Supplementary Material).

The MON810 event was detected in the 2nd batch of diets containing 11 and 33 % of the GM MON810 maize at the DNA and the protein level, while the MON 810 event was not detected in the 2nd batch of control and conventional 2 diets (Table 2). The ratios for the MON810 event level and the Cry1Ab protein content between the diet containing 11 % GMO and the one containing 33 % GMO were 3.1 and 2.6, respectively (Table 2). It should be noted that the detection of the *cry1Ab* gene in the diet sample of the 11 % GM maize failed for any unknown reason, while the MON810 event was quantified and the protein itself was detected as expected.

Following irradiation, microorganisms such as coliforms, *Enterobacteriaceae*, yeast and moulds were not detected in the diets (Table 4, Electronic Supplementary Material).

## Body weight and feed consumption^2^

**Fig. 2:**
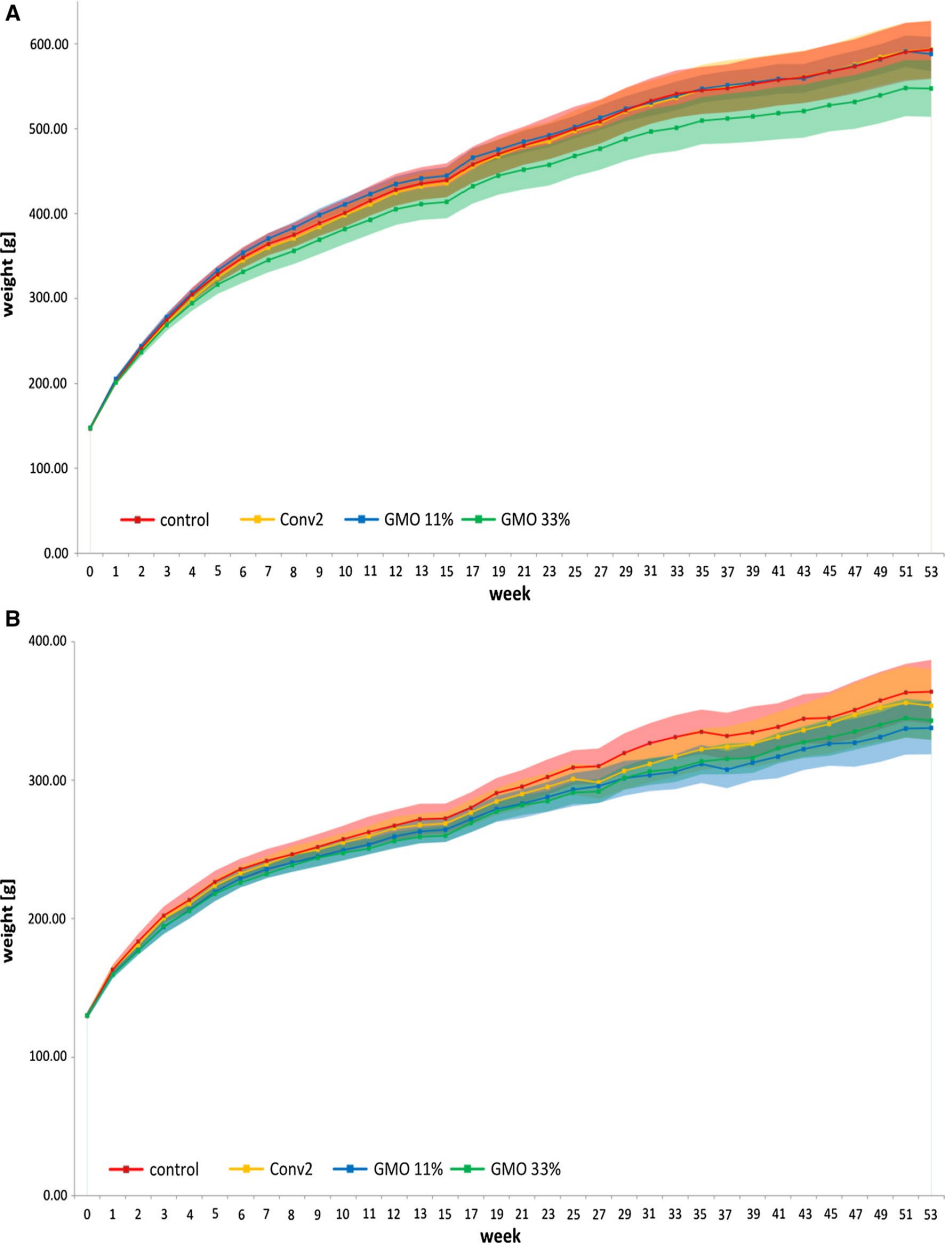
Male and female rat body weights in the 1-year feeding trial.* Line plots* (with confidence intervals) of mean male (**a**) and female (**b**) body weights (g) per cage

The body weight of the male rats in all four groups increased with time (Fig. 2a; Table 5, Electronic Supplementary Material). At the end of the study, the male rats fed the control, conventional 2 and 11 % GMO diet reached a mean body weight of 594, 598 and 592 g, respectively, while those fed the 33 % GMO diet weighed 550 g (Table 5, Electronic Supplementary Material), whereby the difference in the least square means of the body weight between the 33 % GMO diet-fed rats and the control group was not statistically significant. The body weight of the female rats in the four experimental groups also increased time dependently (Fig. 2b; Table 5, Electronic Supplementary Material). At the end of the study, the female rats fed the control, conventional 2, 11 % GMO and 33 % GMO diet reached a mean body weight of 367, 358, 339 and 344 g, respectively (Table 5, Electronic Supplementary Material), whereby the differences of the least square means between the control and the 11 % GMO as well as the 33 % GMO group were not statistically significant.

**Table 5:** Mean values ± standard deviations (SD) of clinical biochemistry parameters in the serum of male and female Wistar Han RCC rats at 3, 6 and 12 months

Parameter	Males				Females			
	Control^1^	Conventional 2^2^	11 % GMO^3^	33 % GMO^4^	Control^5^	Conventional 2^6^	11 % GMO^7^	33 % GMO^8^
At 3 months								
ALP (µkat/l)	1.18 ± 0.12	1.09 ± 0.20	1.10 ± 0.22	1.13 ± 0.19	0.62 ± 0.07	0.61 ± 0.12	0.69 ± 0.06	0.58 ± 0.20
ALT (µkat/l)	0.47 ± 0.08	0.42 ± 0.07	0.46 ± 0.03	0.52 ± 0.09	0.49 ± 0.37	0.34 ± 0.11	0.52 ± 0.22	0.51 ± 0.14
AST (µkat/l)	2.07 ± 0.19	1.67 ± 0.16^b,c^	2.27 ± 0.64	2.50 ± 0.33	2.68 ± 1.02	1.78 ± 0.61	3.06 ± 1.24	3.44 ± 0.75
ALB (g/l)	38.44 ± 2.08	37.91 ± 1.17	39.34 ± 2.61	39.16 ± 2.74	46.64 ± 3.29	45.18 ± 3.53	45.73 ± 0.78	48.47 ± 2.39
TP (g/l)	63.53 ± 1.83	62.80 ± 0.46	64.95 ± 3.45	64.25 ± 2.89	70.07 ± 2.80	67.46 ± 3.69	68.78 ± 1.44	71.76 ± 2.81
GLU (mmol/l)	6.46 ± 0.57	6.54 ± 0.83	6.26 ± 0.87	6.06 ± 0.68	6.62 ± 0.50	6.78 ± 1.40	7.29 ± 0.59	6.10 ± 0.78
CREA (µmol/l)	48.21 ± 6.06	50.29 ± 6.44	48.10 ± 2.56	46.99 ± 4.65	50.72 ± 6.91	54.41 ± 8.42	49.39 ± 3.22	52.28 ± 7.31
U (mmol/l)	6.21 ± 0.59	6.73 ± 0.67	5.88 ± 0.58	5.97 ± 0.48	6.18 ± 0.41	6.31 ± 0.80	6.61 ± 0.42	6.57 ± 0.64
CHOL (mmol/l)	2.03 ± 0.18	2.10 ± 0.13	1.93 ± 0.08	1.99 ± 0.12	1.88 ± 0.33	1.71 ± 0.15	2.13 ± 0.26	1.87 ± 0.14
TRG (mmol/l)	0.80 ± 0.12	1.05 ± 0.22	0.88 ± 0.20	0.80 ± 0.15	0.71 ± 0.16	0.61 ± 0.09	0.57 ± 0.07	0.67 ± 0.14
Ca (mmol/l)	2.50 ± 0.04	2.48 ± 0.04	2.45 ± 0.07	2.44 ± 0.03	2.57 ± 0.06	2.58 ± 0.06	2.56 ± 0.02	2.56 ± 0.02
Cl (mmol/l)	104.80 ± 1.04	104.60 ± 0.96	103.20 ± 0.97	102.60 ± 1.39^a,c^	101.70 ± 2.71	103.10 ± 1.64	100.20 ± 3.58	100.00 ± 1.32
K (mmol/l)	4.80 ± 0.18	4.78 ± 0.19	5.37 ± 1.14	5.50 ± 1.27	5.13 ± 0.86	4.66 ± 0.32	5.02 ± 0.62	5.48 ± 0.84
Na (mmol/l)	144.50 ± 1.46	143.50 ± 0.50	144.10 ± 1.67	144.20 ± 1.92	141.90 ± 3.13	143.40 ± 1.95	141.60 ± 2.22	141.30 ± 1.15
P (mmol/l)	2.08 ± 0.16	2.08 ± 0.20	2.36 ± 0.39	2.43 ± 0.35	2.02 ± 0.41	2.00 ± 0.29	1.91 ± 0.13	1.99 ± 0.27
At 6 months								
ALP (µkat/l)	1.26 ± 0.13	1.42 ± 0.12	1.31 ± 0.23	1.17 ± 0.21	0.63 ± 0.15	0.60 ± 0.16	0.59 ± 0.21	0.53 ± 0.08
ALT (µkat/l)	0.39 ± 0.05	0.43 ± 0.11	0.40 ± 0.03	0.45 ± 0.21	0.59 ± 0.37	0.61 ± 0.26	0.48 ± 0.30	0.58 ± 0.37
AST (µkat/l)	2.42 ± 0.19	3.26 ± 1.21	2.54 ± 0.48^c^	3.38 ± 0.81^c^	3.15 ± 1.60	3.42 ± 1.24	3.13 ± 1.08	3.87 ± 1.45
ALB (g/l)	38.17 ± 2.54	36.83 ± 1.53	38.60 ± 0.64	39.20 ± 1.04	45.18 ± 3.26	46.91 ± 2.83	45.68 ± 1.97	47.17 ± 4.00
TP (g/l)	68.31 ± 3.76	66.91 ± 2.78	68.53 ± 1.40	69.17 ± 1.42	72.59 ± 3.15	75.29 ± 3.15	75.00 ± 2.54	75.53 ± 3.55
GLU (mmol/l)	7.05 ± 1.09	7.89 ± 0.98	6.15 ± 0.40	6.07 ± 1.00	7.85 ± 0.44	7.56 ± 0.64	7.00 ± 0.63	6.07 ± 1.46^a,c^
CREA (µmol/l)	44.38 ± 6.29	42.74 ± 4.79	42.45 ± 3.78	44.72 ± 3.72	41.60 ± 2.55	42.31 ± 2.61	44.29 ± 1.43	48.92 ± 8.42
U (mmol/l)	5.54 ± 0.91	5.84 ± 0.61	5.27 ± 0.12	5.29 ± 0.20	6.39 ± 0.56	5.81 ± 0.51	6.23 ± 0.31	6.11 ± 0.49
CHOL (mmol/l)	2.26 ± 0.19	2.41 ± 0.22	2.21 ± 0.14	2.37 ± 0.19	2.08 ± 0.32	2.11 ± 0.30	2.47 ± 0.21	2.20 ± 0.25
TRG (mmol/l)	1.17 ± 0.36	1.10 ± 0.07	1.12 ± 0.18	1.08 ± 0.21	0.71 ± 0.17	0.72 ± 0.06	0.58 ± 0.15	0.58 ± 0.23
Ca (mmol/l)	2.63 ± 0.07	2.61 ± 0.11	2.62 ± 0.04	2.62 ± 0.09	2.57 ± 0.07	2.59 ± 0.05	2.59 ± 0.04	2.54 ± 0.05
Cl (mmol/l)	103.90 ± 1.39	101.40 ± 1.29^a,c^	102.80 ± 0.76	103.10 ± 1.75	100.90 ± 1.39	99.70 ± 2.36	100.50 ± 2.06	100.30 ± 2.56
K (mmol/l)	4.97 ± 0.44	5.16 ± 0.18	5.24 ± 0.25	5.63 ± 0.64	4.90 ± 0.48	4.94 ± 0.22	5.15 ± 0.70	5.36 ± 0.98
Na (mmol/l)	146.30 ± 1.30	144.20 ± 1.04^a,c^	146.80 ± 0.57	146.50 ± 0.79	143.70 ± 1.79	144.40 ± 1.98	143.30 ± 2.51	144.80 ± 2.31
P (mmol/l)	1.65 ± 0.20	1.51 ± 0.27	2.05 ± 0.28^a,c^	2.01 ± 0.23^c^	1.36 ± 0.19	1.37 ± 0.07	1.64 ± 0.22	1.87 ± 0.47
At 12 months								
ALP (µkat/l)	1.31 ± 0.24	1.23 ± 0.20	1.42 ± 0.23	1.17 ± 0.23	0.59 ± 0.25	0.51 ± 0.07	0.56 ± 0.13	0.53 ± 0.14
ALT (µkat/l)	0.80 ± 0.34	0.75 ± 0.34	0.71 ± 0.11	0.70 ± 0.21	0.68 ± 0.17	0.79 ± 0.33	0.61 ± 0.16	0.86 ± 0.34
AST (µkat/l)	2.58 ± 0.79	2.48 ± 0.63	2.50 ± 0.49	2.76 ± 0.62	2.46 ± 0.79	2.69 ± 0.71	2.39 ± 0.57	3.36 ± 1.06^a^
ALB (g/l)	40.04 ± 1.94	39.46 ± 1.65	40.52 ± 1.47	39.76 ± 1.94	42.62 ± 2.07	42.85 ± 3.01	43.62 ± 3.40	42.77 ± 2.48
TP (g/l)	69.39 ± 1.93	68.64 ± 2.45	69.75 ± 1.57	68.06 ± 2.50	71.69 ± 2.70	71.92 ± 2.70	72.70 ± 3.90	70.14 ± 2.92
GLU (mmol/l)	6.28 ± 1.13	6.17 ± 0.75	5.32 ± 0.49^c^	5.65 ± 1.08	5.47 ± 0.51	5.77 ± 0.80	5.51 ± 0.95	4.91 ± 0.65
CREA (µmol/l)	42.53 ± 4.38	40.31 ± 3.53	42.03 ± 3.24	39.40 ± 4.37	41.50 ± 2.96	42.07 ± 4.30	44.91 ± 2.66^b,c^	42.85 ± 4.61
U (mmol/l)	5.24 ± 0.71	5.52 ± 0.51	5.20 ± 0.69	5.12 ± 0.69	5.31 ± 0.67	4.81 ± 0.32	5.58 ± 0.66	5.34 ± 0.38
CHOL (mmol/l)	2.76 ± 0.34	2.69 ± 0.37	2.55 ± 0.29	2.73 ± 0.49	2.20 ± 0.39	2.46 ± 0.52	2.55 ± 0.42	2.24 ± 0.29
TRG (mmol/l)	1.44 ± 0.43	1.57 ± 0.64	1.41 ± 0.54	1.45 ± 0.46	1.05 ± 0.29	1.01 ± 0.30	0.98 ± 0.28	0.90 ± 0.23
Ca (mmol/l)	2.66 ± 0.07	2.66 ± 0.10	2.64 ± 0.06	2.61 ± 0.05	2.60 ± 0.03	2.61 ± 0.04	2.62 ± 0.07	2.59 ± 0.04
Cl (mmol/l)	105.70 ± 2.50	105.30 ± 2.12	104.45 ± 1.54	103.70 ± 2.24	101.90 ± 1.91	101.40 ± 1.91	102.55 ± 1.50	102.95 ± 1.54
K (mmol/l)	5.41 ± 0.27	5.53 ± 0.49	5.76 ± 0.51	5.54 ± 0.31	4.71 ± 0.23	4.41 ± 0.20^b,c^	4.67 ± 0.50	4.88 ± 0.64
Na (mmol/l)	145.50 ± 2.17	145.15 ± 1.49	145.80 ± 1.23	143.65 ± 1.72	141.80 ± 1.09	142.45 ± 1.54	142.35 ± 1.08	142.70 ± 1.55
P (mmol/l)	1.89 ± 0.19	1.98 ± 0.26	2.12 ± 0.15^b,c^	1.95 ± 0.21	1.52 ± 0.25	1.46 ± 0.31	1.57 ± 0.27	1.63 ± 0.32

*ALP* alkaline phosphatase, *ALT* alanine aminotransferase, *AST* aspartate aminotransferase, *ALB* albumin, *TP* total protein, *GLU* glucose, *CREA* creatinine, *U* urea, *CHOL* cholesterol, *TRG* triglycerides, *Ca* calcium, *Cl* chloride, *K* potassium, *Na* sodium, *P* phosphorus

^1^10, 10 and 20 male rats fed the diet containing 33 % near-isogenic non-GM maize for 3, 6 or 12 months, respectively, were analysed

^2^10, 10 and 20 male rats fed the diet containing 33 % conventional 2 maize for 3, 6 or 12 months, respectively, were analysed

^3^10 (9 in the case of ALT), 10 and 20 (18 in the case of Alb, TP and K) male rats fed the diet containing 11 % GMO maize for 3, 6 or 12 months, respectively, were analysed

^4^9 (8 in the case of ALT), 10 (9 in the case of ALT) and 19 (17 in the case of ALB, TP and K) male rats fed the diet containing 33 % GMO maize for 3, 6 or 12 months, respectively, were analysed

^5^10, 10 and 19 female rats fed the diet containing 33 % near-isogenic non-GM maize for 3, 6 or 12 months, respectively, were analysed

^6^10 (9 in the case of CHOL), 10 and 20 female rats fed the diet containing 33 % conventional 2 maize for 3, 6 or 12 months, respectively, were analysed

^7^10 (9 in the case of ALT), 10 and 19 (17 in the case of ALP) female rats fed the diet containing 11 % GMO maize for 3, 6 or 12 months, respectively, were analysed

^7^10 (9 in the case of ALP), 10 (9 in the case of ALP) and 20 (19 in the case of AST, TP, CHOL, TRG, Ca, K and P, 18 in the case of ALP, ALT and ALB) female rats fed the diet containing 33 % GMO maize for 3, 6 or 12 months, respectively, were analysed

^a^Statistically significant difference to the control value based on one-way ANOVA and post hoc *t* test (*p* ≤ 0.05) as well as with the Dunnett test (*p* ≤ 0.05)

^(a)^Statistically significant difference to the control value (*p* < 0.05) based on one-way ANOVA and post hoc *t* test, post hoc Dunnett test not significant

^b^Statistically significant difference to the control value (*p* < 0.05) based on the Wilcoxon test

^c^Statistically significant difference to the control value based on the 95 % confidence interval of the SES

**Fig. 3:**
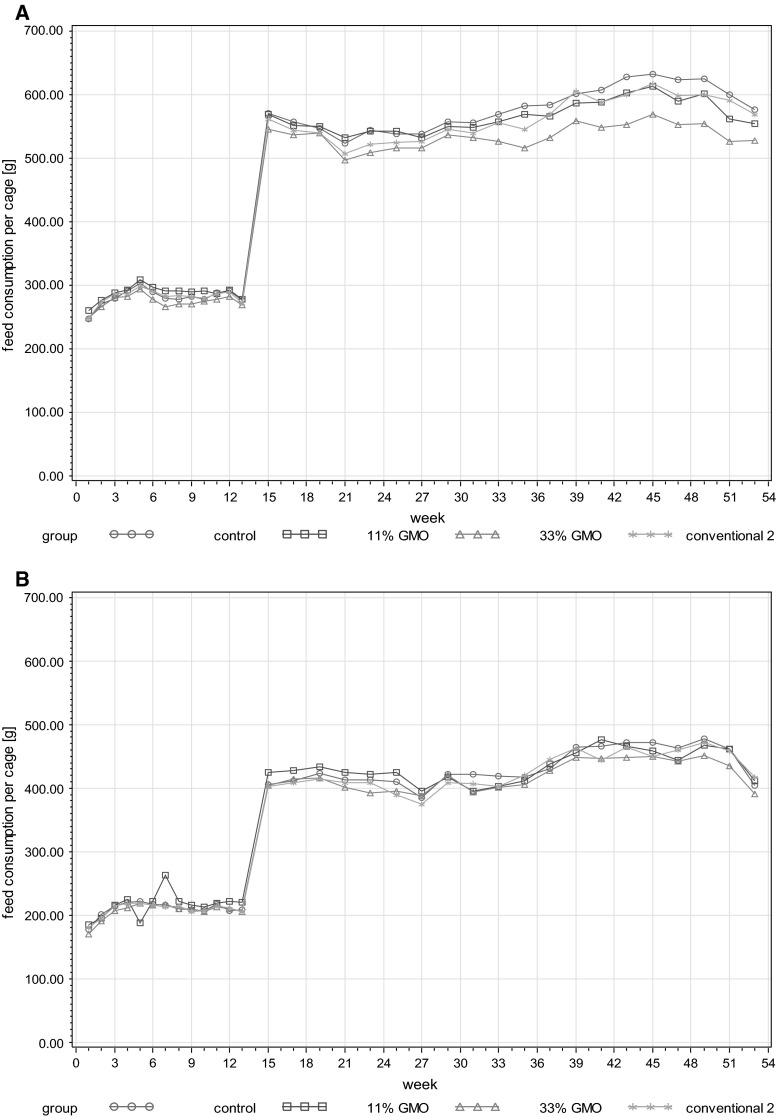
Male and female rat feed consumption in the 1-year feeding trial.* Line plots* of mean feed consumption per cage (g) and per week (weeks 1–13) or per 2 weeks (weeks 15–53) in the case of male (**a**) and female rats (**b**)

Feed consumption in male rats increased in the first 5 weeks, remained relatively constant until week 31 and slightly increased until week 49 (Fig. 3a; Table 6, Electronic Supplementary Material). Males being fed the conventional 2, 11 % GMO or 33 % GMO diet consumed less feed (in the order 33 % GMO <11 % GMO <conventional 2) than the males in the control group (Table 6, Electronic Supplementary Material). Feed consumption in female rats increased in the first 4 weeks, remained relatively constant until week 35 and then slightly increased until week 39 (Fig. 3b; Table 6, Electronic Supplementary Material). Feed consumption was similar in rats fed the control, the conventional 2 or the 11 % GMO diet, while it was slightly lower in female rats fed the 33 % GMO diet (Table 6, Electronic Supplementary Material). The differences of the least square means in feed consumption between the control and the 11 % GMO, 33 % GMO and conventional 2 groups in male and female rats were not statistically significant.

**Table 6:** Urinalysis data of male rats fed the control, conventional 2, 11 % GMO or 33 % GMO diet for 3, 6 and 12 months

Point in time (months)	Number of animals	Protein (g/L)				Ketones (mmol/L)				Nitrate (µmol/L)		Leucocytes (leucocytes/µL)				Erythrocytes (erythrocytes/µL)				
		0	0.25	0.75	1.5	0	0.5	1.5	5	0	11	0	25	100	500	0	10	25	50	250
Control																				
3	10	10	0	0	0	1	0	7	2	10	0	10	0	0	0	7	2	1	0	0
6	10	10	0	0	0	4	0	5	1	10	0	1	7	2	0	9	0	1	0	0
12	10	8	1	0	1	1	2	6	1	10	0	4	5	0	1	9	0	0	0	1
Conv. 2																				
3	10	9	1	0	0	0	3	6	1	10	0	8	2	0	0	10	0	0	0	0
6	10	9	1	0	0	3	3	4	0	10	0	0	5	4	1	10	0	0	0	0
12	10	8	1	0	1	1	4	5	0	10	0	5	3	1	1	8	1	0	0	1
11 % GMO																				
3	10	10	0	0	0	0	0	10	0	10	0	10	0	0	0	8	1	1	0	0
6	10	8	2	0	0	0	5	4	1	10	0	0	7	3	0	10	0	0	0	0
12	10	6	2	1	1	0	1	9	0	10	0	2	5	1	2	10	0	0	0	0
33 % GMO																				
3	10	10	0	0	0	2	3	5	0	9	1	8	2	0	0	8	2	0	0	0
6	10	10	0	0	0	5	2	3	0	10	0	1	7	1	1	9	1	0	0	0
12	10	7	2	0	1	0	3	7	0	10	0	3	4	1	2	8	1	0	1	0

Point in time (months)	Number of animals	pH					Osmolality (mOsm/kg H_2_O)
		5	6	6.5	7	8	
Control							
3	10	1	2	6	1	0	357.1 ± 122.7
6	10	2	2	5	1	0	398.6 ± 109.7
12	10	1	2	5	2	0	564.1 ± 191.5
Conv. 2							
3	10	0	1	7	2	0	441.7 ± 219.6
6	10	5	1	3	1	0	518.5 ± 152.6
12	10	1	4	5	0	0	501.2 ± 88.7
11 % GMO							
3	10	0	0	4	6	0	407.4 ± 134.2
6	10	0	5	2	3	0	463.2 ± 137.7
12	10	2	6	1	1	0	604.3 ± 211.8
33 % GMO							
3	10	0	0	2	7	1	488.4 ± 274.8
6	10	0	3	2	4	1	540.0 ± 269.8
12	10	1	2	2	5	0	589.9 ± 213.9

Glucose, bilirubin and urobilinogen were negative in all analysed urine samples

**Fig. 4:**
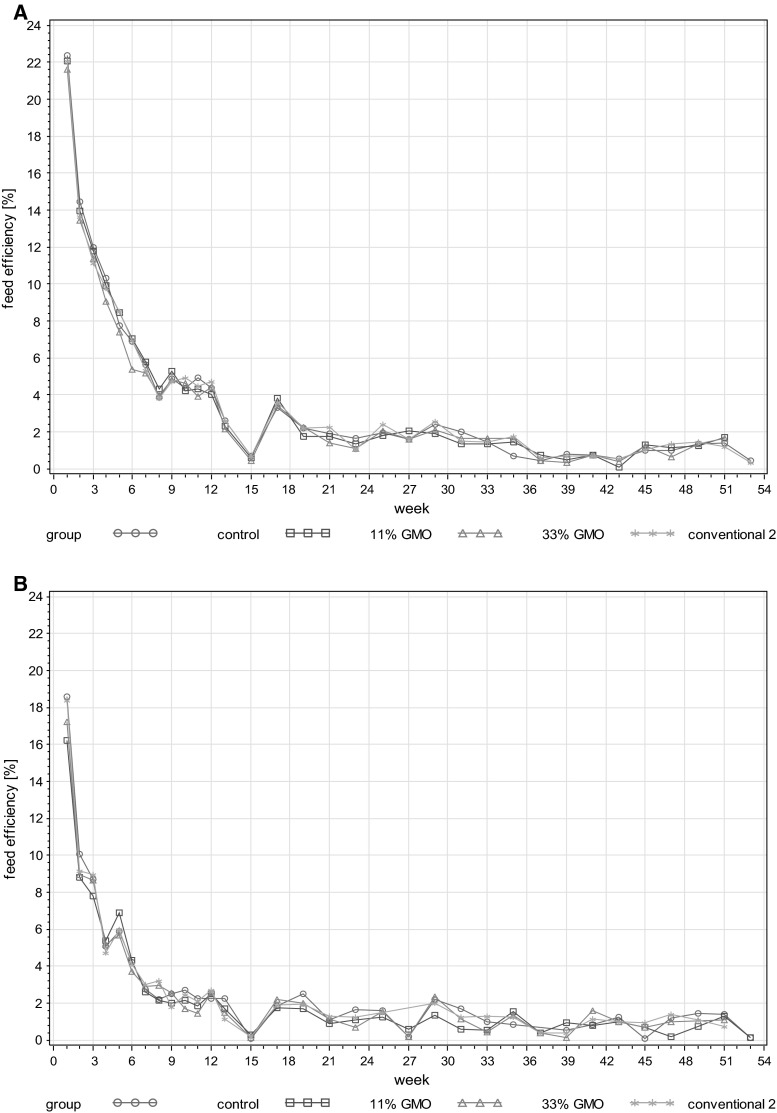
Male and female rat feed efficiency in the 1-year feeding trial.* Line plots* of mean feed efficiency per cage (g) and per week (weeks 1–13) or per 2 weeks (weeks 15–53) in the case of male (**a**) and female rats (**b**)

Feed efficiency in the case of male rats was very similar in all four experimental groups (Fig. 4a; Table 7, Electronic Supplementary Material), and the same holds true for female rats (Fig. 4b; Table 7, Electronic Supplementary Material). No statistically significant differences between the least square means were observed among the different experimental groups in male as well as in female rats.

**Table 7:** Urinalysis data of female rats fed the control, conventional 2, 11 % GMO or 33 % GMO diet for 3, 6 and 12 months

Point in time (months)	Number of animals	Protein (g/L)				Ketones (mmol/L)				Leucocytes (leucocytes/µL)				Erythrocytes (erythrocytes/µL)					pH				
		0	0.25	0.75	1.5	0	0.5	1.5	5	0	25	100	500	0	10	25	50	250	5	6	6.5	7	8
Control																							
3	10	10	0	0	0	2	3	5	0	9	1	0	0	10	0	0	0	0	2	3	5	0	0
6	10	10	0	0	0	6	3	1	0	10	0	0	0	10	0	0	0	0	0	7	2	1	0
12	9	8	1	0	0	2	1	6	0	3	3	3	0	9	0	0	0	0	0	8	1	0	0
Conv. 2																							
3	10	9	1	0	0	3	4	3	0	8	2	0	0	10	0	0	0	0	0	5	2	3	0
6	10	9	0	0	1	4	6	0	0	7	2	0	1	10	0	0	0	0	0	5	2	3	0
12	10	8	2	0	0	7	1	2	0	7	1	2	0	10	0	0	0	0	2	5	1	2	0
11 % GMO																							
3	10	10	0	0	0	3	5	2	0	9	1	0	0	10	0	0	0	0	5	2	3	0	0
6	10	10	0	0	0	8	0	2	0	9	1	0	0	10	0	0	0	0	1	8	1	0	0
12	10	7	3	0	0	3	3	4	0	6	1	1	2	9	0	0	0	1	4	4	0	2	0
33 % GMO																							
3	10	9	1	0	0	3	3	4	0	9	1	0	0	10	0	0	0	0	0	5	5	0	0
6	10	9	1	0	0	4	1	5	0	5	2	3	0	9	0	0	0	1	0	7	3	0	0
12	10	7	1	2	0	3	2	5	0	5	3	1	1	9	0	0	1	0	4	5	1	0	0

Point in time (months)	Number of animals	Osmolality (mOsm/kg H_2_O)
Control		
3	10	484.5 ± 120.2
6	10	432.0 ± 105.6
12	9	588.6 ± 142.9
Conv. 2		
3	10	402.6 ± 190.9
6	10	478.9 ± 206.7
12	10	465.0 ± 219.5
11 % GMO		
3	10	444.1 ± 220.0
6	10	463.2 ± 137.7
12	10	604.3 ± 211.8
33 % GMO		
3	10	488.4 ± 274.8
6	10	540.0 ± 269.8
12	10	589.9 ± 213.9

Glucose, bilirubin, urobilinogen and nitrate were negative in all analysed urine samples

## Clinical and ophthalmological observations

The following three rats had to be killed before the end of the feeding trial: (1) rat No. 15: a male rat fed the 33 % GMO diet was killed on day 227 due to a paraplegia of the hind limbs that led to strong loss of weight; (2) rat No. 131: a female rat fed the 11 % GMO diet was killed on day 244 due to the development of a yolk sac carcinoma localized in the right ovary that led to multiple metastases in the abdominal cavity; and (3) rat No. 159: a female rat fed the control diet was killed day 327 due to the development of a mammary gland comedocarcinoma. A very limited number of clinical signs were observed in the rest of the animals (Table 3). The ophthalmological analyses revealed no alterations in all four experimental groups immediately before starting the feeding trial as well as at the end of the study.

## Haematology, clinical biochemistry and urine analyses^3^

**Fig. 5:**
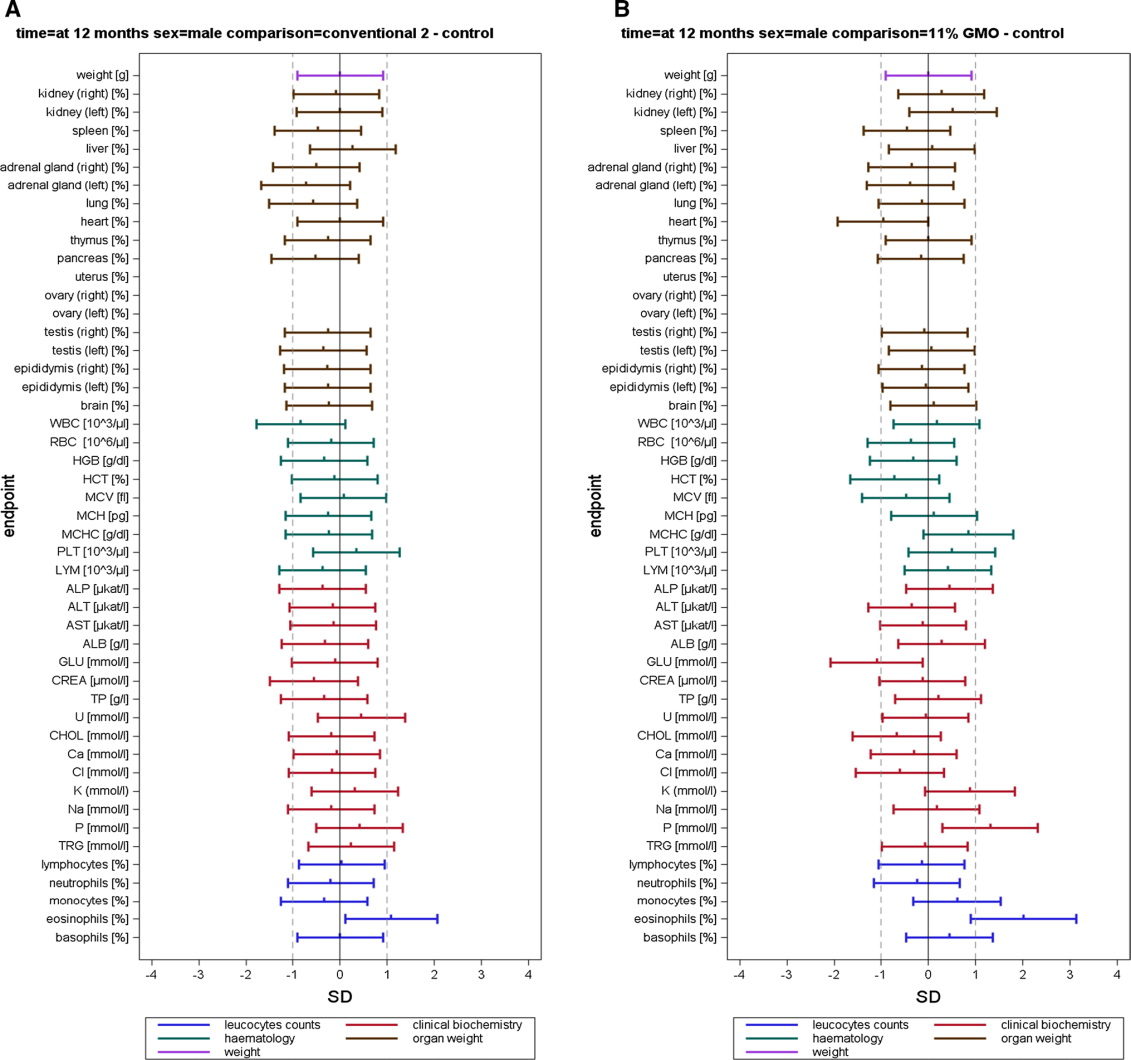

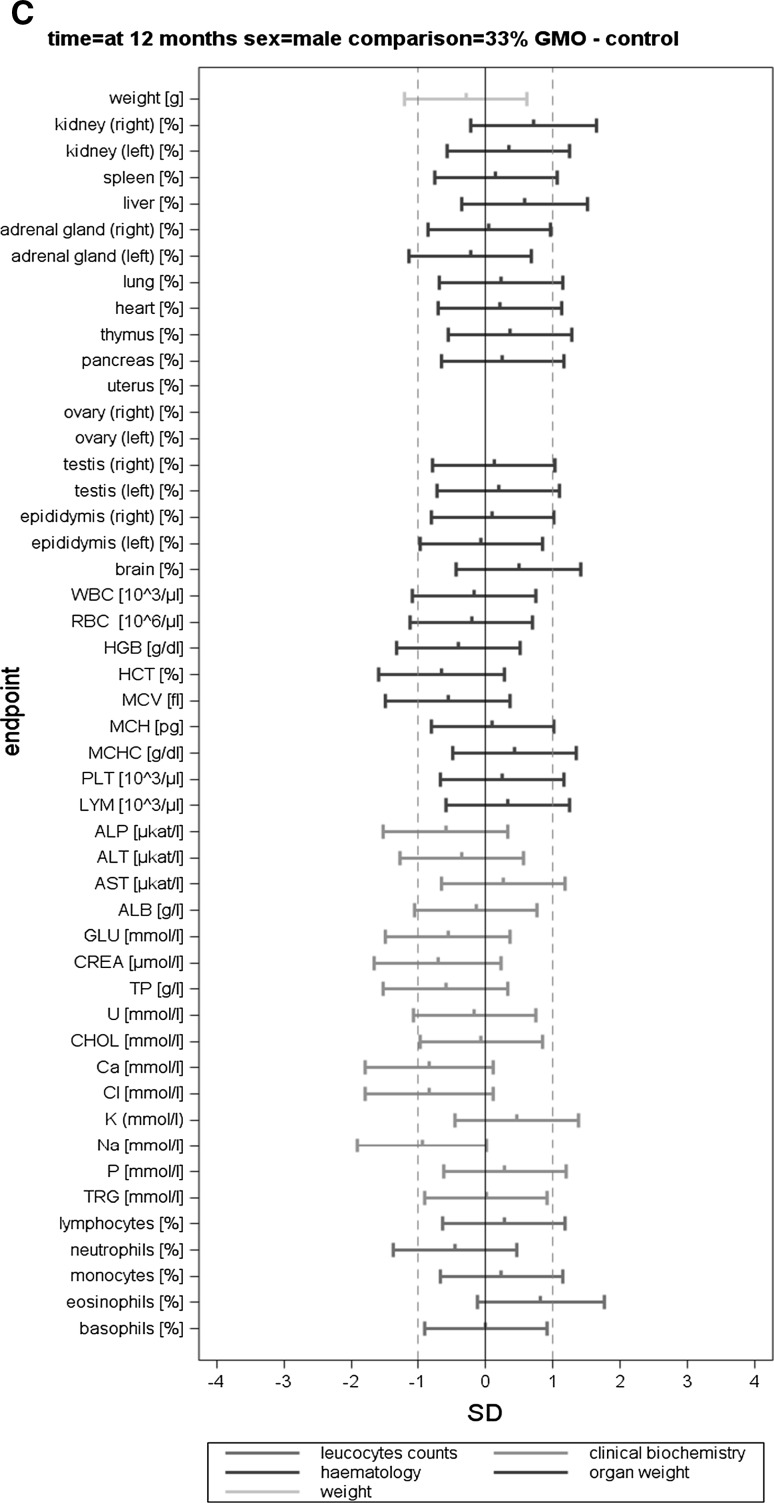
Standardized effect size graphs for the comparison of the body weight, relative organ weight, haematology, clinical biochemistry and differential leucocyte count data between the control and the conventional 2 (A), 11 % GMO (B) and 33 % GMO (C) groups in the case of male rats in the 1-year feeding trial

**Fig. 6:**
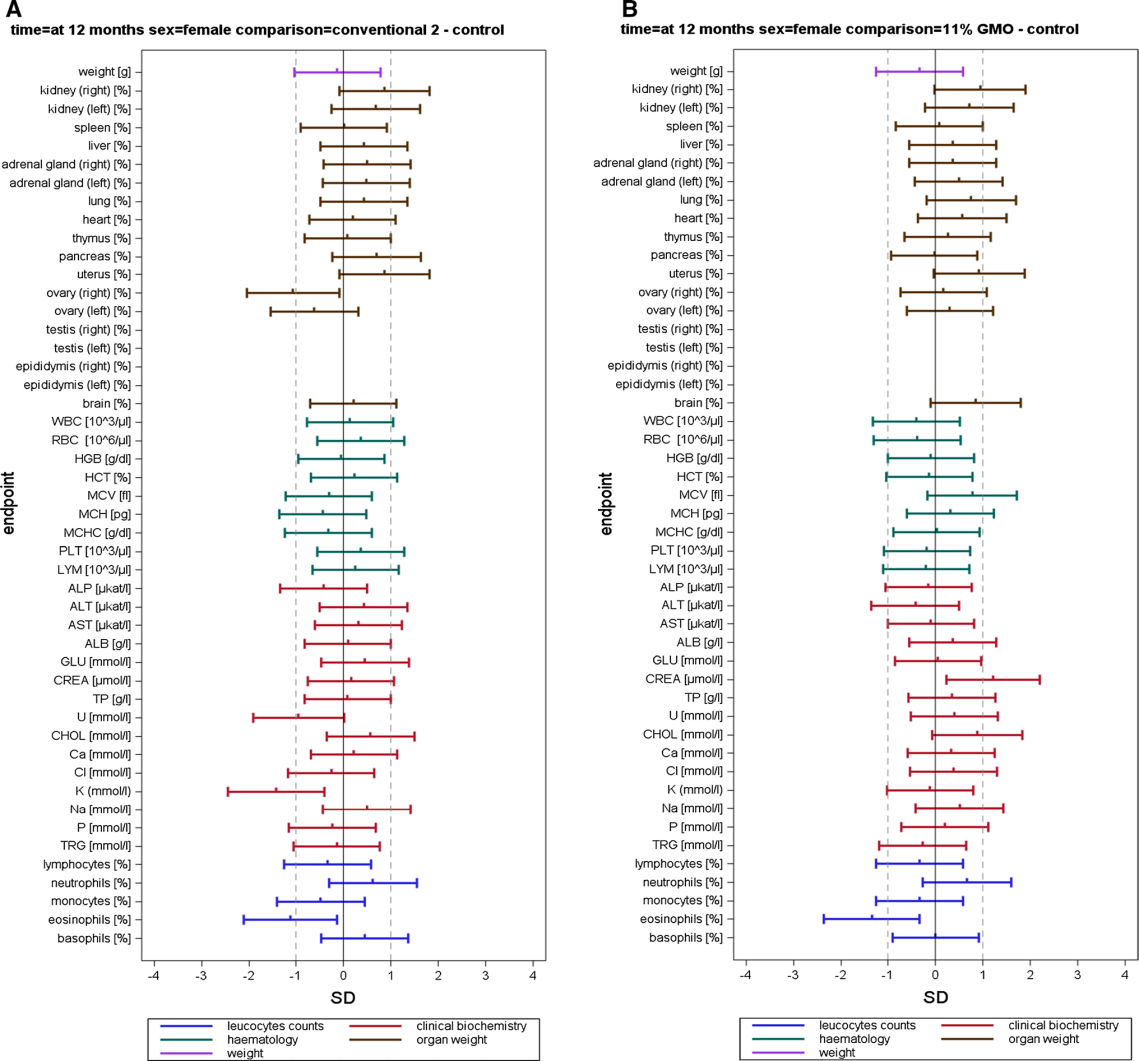

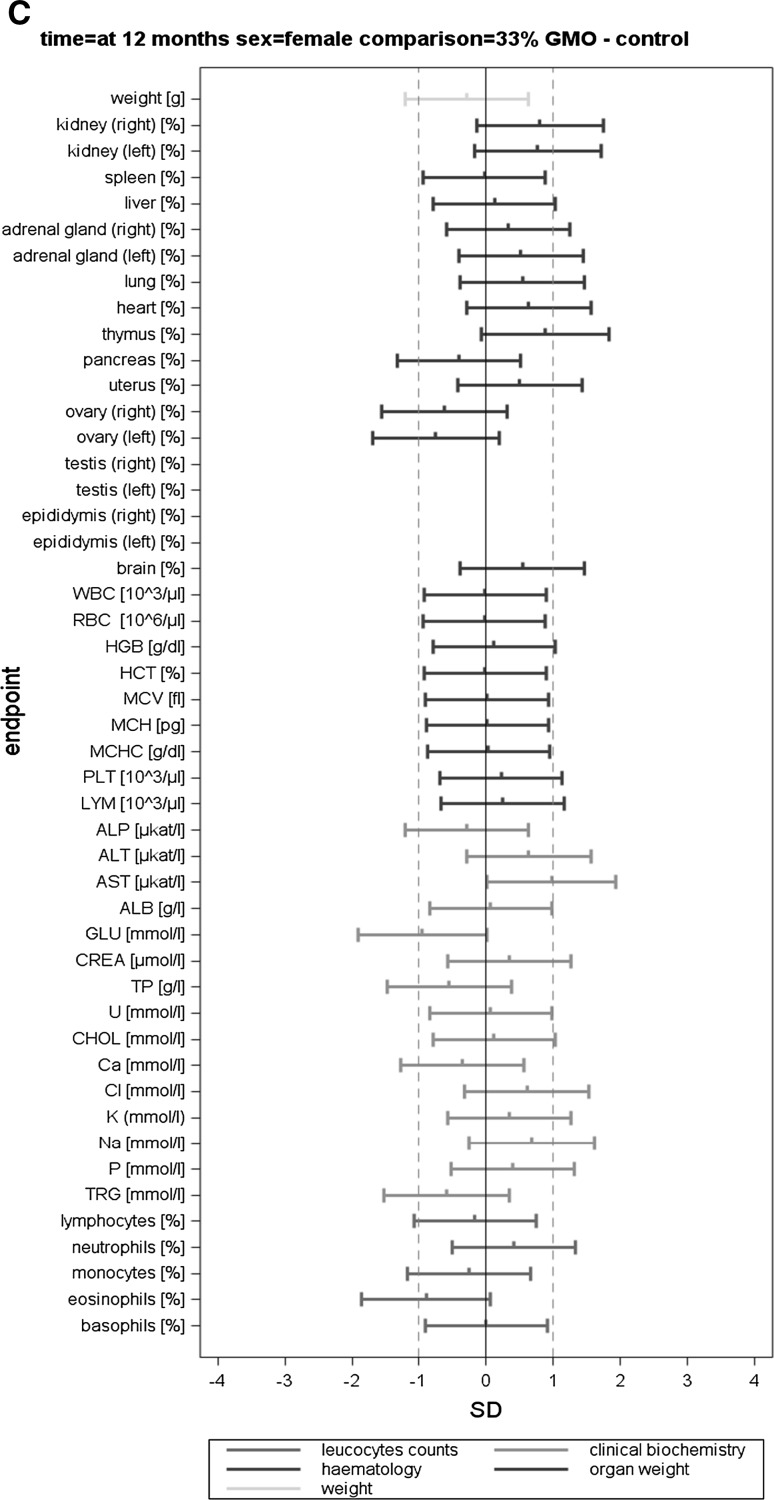
Standardized effect size graphs for the comparison of the body weight, relative organ weight, haematology, clinical biochemistry and differential leucocyte count data between the control and the conventional 2 (**a**), 11 % GMO (**b**) and 33 % GMO (**c**) groups in the case of female rats in the 1-year feeding trial

The haematology parameters measured in the blood samples of male and female rats at 3, 6 and 12 months are shown in Table 4 and the corresponding SES graphs with the data at *t* = 12 months in Figs. 5a–c and 6a–c.

After 3 months, no statistically significant differences were observed when the haematology parameters WBC, RBC, HGB, HCT, MCV, MCH, MCHC, PLT and LYM of the control, conventional 2, 11 % GMO and 33 % GMO groups were compared, this being the case of male as well as female rats (Table 4). The differential leucocyte count showed that the percentage of eosinophils was significantly higher in female rats fed the 33 % GMO diet than in the animals fed the control diet (Table 4).

After 6 months, no statistically significant differences were observed when the haematology parameters and the differential leucocyte count of male and female rats fed the control, conventional 2, 11 % GMO and 33 % GMO diets were compared, except for the percentage of white blood cells, which was significantly higher in male rats fed the 11 % GMO and the 33 % GMO diets, and the percentage of eosinophils, which was significantly lower in female rats fed the 33 % GMO diet when compared to the corresponding animals fed the control diet (Table 4).

After 12 months, no statistically significant differences were observed when the haematology parameters and the differential leucocyte count of male and female rats fed the control, conventional 2, 11 % GMO and 33 % GMO diets were compared, except for the percentage of eosinophils, which was increased in male as well as in female rats fed the conventional 2 and the 11 % GMO diets when compared to the corresponding animals fed the control diet (Table 4; Figs. 5a, b, 6a, b).

With the classical statistical methods, an increase in the number of WBC after 6 months was additionally observed in female rats of the conventional 2 group if compared to the control group (Table 4).

The clinical biochemistry parameters measured in serum of male and female rats are shown in Table 5 and the corresponding SES graphs with the data at *t* = 12 months in Figs. 5a–c and 6a–c. After 3 months, no statistically significant differences were observed when all biochemical parameters of male and female rats fed the control, conventional 2, 11 % GMO and 33 % GMO diets were compared, except for the AST activity in male rats fed the conventional 2 diet and the Cl levels in male rats fed the 33 % GMO diet, which were significantly decreased when compared to male rats fed the control diet (Table 5).

After 6 months, the AST activity was significantly increased in male rats fed the 11 % GMO diet, the P level was significantly increased in male rats fed the 33 % GMO diet, and the Cl and Na levels were significantly decreased in male rats fed the conventional 2 diet if compared to the male rats fed the control diet (Table 5). No statistically significant differences were observed when all measured biochemical parameters of female rats in all four experimental groups were compared, except for the GLU level, which was significantly lower in female animals fed the 33 % GMO diet than in those fed the control diet (Table 5).

After 12 months, the GLU level was significantly reduced and the P level significantly increased in male rats fed the 11 % GMO diet if compared to those fed the control diet (Table 5; Fig. 5b). No statistically significant differences were observed when all measured biochemical parameters of female rats in all four experimental groups were compared, except for the CREA level, which was significantly higher in animals fed the 11 % GMO diet, and the K level, which was significantly lower in animals fed the conventional 2 diet than in those fed the control diet (Table 5; Fig. 6a, b).

With the classical statistical methods, an increase in the AST activity was additionally observed in female rats fed the 33 % GMO diet for 12 months when compared to the corresponding control group (Table 5).

Urinalysis data of male rats fed the control, conventional 2, 11 % GMO or 33 % GMO diet for 3, 6 and 12 months are shown in Table 6, while the urinalysis data of female rats fed the control, conventional 2, 11 % GMO or 33 % GMO diet for 3, 6 and 12 months are shown in Table 7. In male rats, the semi-quantitative evaluation of protein, ketones, nitrate, leucocytes and erythrocytes did not reveal major differences between the four experimental groups, while a tendency to a higher pH was observed in urine samples from rats fed the 11 % GMO and 33 % GMO diets if compared to those of animals fed the control and the conventional 2 diets (Table 6). Moreover, osmolality increased with age and to a similar extent in all four experimental groups. In female rats, the semi-quantitative evaluation of protein, ketones, nitrate, leucocytes and erythrocytes as well as the pH determination did not reveal major differences between the four experimental groups, whereas osmolality increased with age and to a similar extent in all four experimental groups (Table 7).

## Relative organ weights, gross necropsy and histopathology^2,^^4^

No statistically significant differences were observed when the relative weight of all organs in male rats fed the control, conventional 2, 11 % GMO and 33 % GMO diets was compared (Table 8). In female rats fed the conventional 2 diet, the relative weight of the right ovary was significantly lower than in control female rats, whereas no statistically significant differences were observed when the relative weight of all other organs in the female animals of all four experimental groups was compared (Table 8). The weight of the pair organs (i.e. the kidneys, adrenal glands, testes and epididymides in male rats, the kidneys, adrenal glands and ovaries in the female rats) was also analysed as a grouped value, as recommended by Sellers et al. (2007), and no statistically significant differences between the groups were observed (data not shown).

**Table 8:** Relative weight of the organs (cage mean ± SD) of male and female Wistar Han RCC rats in the 1-year feeding trial with the GM maize MON810

Male rats	Relative organ weights (organ weight/body weight × 100)^1^			
Organ	Control	Conventional 2	11 % GMO	33 % GMO
Kidney (right)	0.234 ± 0.018	0.233 ± 0.017	0.239 ± 0.016	0.245 ± 0.012
Kidney (left)	0.219 ± 0.015	0.219 ± 0.016	0.228 ± 0.020	0.224 ± 0.016
Spleen	0.182 ± 0.020	0.175 ± 0.009	0.174 ± 0.016	0.186 ± 0.029
Liver	2.370 ± 0.122	2.410 ± 0.174	2.380 ± 0.144	2.466 ± 0.199
Adrenal gland (right)	0.004 ± 0.0003	0.004 ± 0.001	0.004 ± 0.001	0.004 ± 0.001
Adrenal gland (left)	0.004 ± 0.001	0.004 ± 0.001	0.004 ± 0.001	0.004 ± 0.001
Lung	0.247 ± 0.020	0.235 ± 0.022	0.244 ± 0.018	0.251 ± 0.020
Heart	0.207 ± 0.010	0.207 ± 0.009	0.197 ± 0.011	0.209 ± 0.012
Thymus	0.095 ± 0.018	0.091 ± 0.014	0.095 ± 0.019	0.101 ± 0.012
Pancreas	0.124 ± 0.023	0.114 ± 0.015	0.121 ± 0.013	0.129 ± 0.021
Testis (right)	0.370 ± 0.038	0.355 ± 0.072	0.367 ± 0.019	0.374 ± 0.036
Testis (left)	0.366 ± 0.035	0.346 ± 0.067	0.367 ± 0.016	0.373 ± 0.041
Epididymis (right)	0.117 ± 0.013	0.112 ± 0.019	0.115 ± 0.009	0.118 ± 0.011
Epididymis (left)	0.117 ± 0.011	0.112 ± 0.020	0.116 ± 0.007	0.116 ± 0.012
Brain	0.391 ± 0.037	0.383 ± 0.036	0.394 ± 0.023	0.410 ± 0.040

Female rats	Relative organ weights (organ weight/body weight × 100)^2^			
Organ	Control	Conventional 2	11 % GMO	33 % GMO
Kidney (right)	0.280 ± 0.032	0.306 ± 0.027	0.307 ± 0.025	0.300 ± 0.015
Kidney (left)	0.262 ± 0.025	0.278 ± 0.024	0.282 ± 0.030	0.278 ± 0.018^b^
Spleen	0.227 ± 0.041	0.227 ± 0.036	0.229 ± 0.028	0.226 ± 0.027
Liver	2.559 ± 0.235	2.667 ± 0.268	2.639 ± 0.203	2.583 ± 0.149
Adrenal gland (right)	0.009 ± 0.001	0.009 ± 0.001	0.009 ± 0.001	0.009 ± 0.001
Adrenal gland (left)	0.009 ± 0.002	0.010 ± 0.002	0.010 ± 0.003	0.010 ± 0.002
Lung	0.305 ± 0.027	0.317 ± 0.030	0.326 ± 0.028	0.318 ± 0.024
Heart	0.248 ± 0.021	0.252 ± 0.017	0.259 ± 0.018	0.259 ± 0.012
Thymus	0.119 ± 0.019	0.120 ± 0.013	0.123 ± 0.012	0.137 ± 0.022
Pancreas	0.185 ± 0.024	0.201 ± 0.022	0.184 ± 0.025	0.176 ± 0.023
Uterus	0.177 ± 0.051	0.219 ± 0.046	0.235 ± 0.075	0.203 ± 0.055
Ovary (right)	0.012 ± 0.003	0.009 ± 0.003^a,c^	0.013 ± 0.003	0.011 ± 0.003
Ovary (left)	0.012 ± 0.003	0.009 ± 0.003	0.012 ± 0.003	0.010 ± 0.001
Brain	0.600 ± 0.052	0.612 ± 0.063	0.646 ± 0.057	0.627 ± 0.052

^1^The organs of 20 (19 in the case of the right adrenal gland), 20, 20 and 19 male rats fed the control, conventional 2, 11 % GMO or 33 % GMO diet, respectively, were evaluated

^2^The organs of 19, 20, 19 and 20 female rats fed the control, conventional 2, 11 % GMO or 33 % GMO diet, respectively, were evaluated

^a^Statistically significant difference to control group based on one-way ANOVA, post hoc *t* test (*p* < 0.05), post hoc Dunnett test

(*p* < 0.05)

^(a)^Statistically significant difference to control group (*p* < 0.05) based on one-way ANOVA and post hoc *t* test, post hoc Dunnett test not significant

^b^Statistically significant difference to control group (*p* < 0.05) based on Wilcoxon test

^c^Statistically significant difference to control group based on 95 % confidence interval of the SES

With the classical statistical methods, the relative left kidney weight of female rats fed the 33 % GMO diet was additionally increased when compared to the corresponding control group value (Table 8).

The gross necropsy observations and histopathological findings in all male rats having been fed the control and the 33 % GMO diets are shown in Table 9, while those of all female animals having been fed the control and the 33 % GMO diets are shown in Table 10. Five out of 20 male rats fed the control diet and 1 out of 20 male rats fed the 33 % GMO diet showed macroscopic alterations, which were not accompanied by corresponding histopathological alterations when analysed under the light microscope (Table 9). Eleven out of 20 female rats fed the control diet showed macroscopic alterations: in 1 out of the 11 animals showing macroscopic alterations, no histopathological alterations were detected (Table 10). Sixteen out of 20 female rats fed the 33 % GMO diet showed macroscopic alterations: in 5 out of the 16 animals showing macroscopic alterations, no histopathological alterations were observed (Table 10). Moreover, as stated in the OECD Test Guideline 452 (OECD 2009), all tissues showing macroscopic abnormalities in the other experimental groups should also be analysed histopathologically. These data are shown for the animals having been fed the conventional 2 diet in Table 8 of the Electronic Supplementary Material and for those having been fed the 11 % GMO diet in Table 9 of the Electronic Supplementary Material.

**Table 9:** Gross necropsy observations and histopathological findings in male Wistar Han RCC rats fed the control or 33 % GMO diet for 1 year

Group	Animal no.	Gross necropsy observations	Histopathological findings
Control	41	None	No histopathological alterations
	42	None	No histopathological alterations
	43	None	Focal interstitial nephritis in the left and right kidney, lymphoepithelioid granuloma in the small intestine
	44	None	No histopathological alterations
	45	None	No histopathological alterations
	46	Red-brown coloured surface on the right side of the prostate	No histopathological alterations
	47	Thickened area on the frontal bone	Bone sample not analysed
	48	None	Meibomian gland hyperplasia
	49	None	Focal mononuclear infiltration in the heart
	50	None	No histopathological alterations
	51	Brown coloured, oval-shaped mass of soft consistency in adipose tissue surrounding the left epididymis	No histopathological alterations
	52	None	Focal mononuclear infiltration in the heart, focal chronic interstitial prostatitis and lymphatic sinus ectasia
	53	None	No histopathological alterations
	54	Bilateral petechial haemorrhage (site: submandibular lymph node)	Focal mononuclear infiltration in the heart, no further histopathological alterations
	55	None	No histopathological alterations
	56	None	Tubular dilation and atrophy in the left and right kidney
	57	Grey-coloured adipose tissue surrounding the right epididymis	No histopathological alterations
	58	None	No histopathological alterations
	59	None	Cysts in the pituitary gland
	60	None	No histopathological alterations

Group	Animal no.	Gross necropsy observations	Histopathological findings
33 % GMO	1	None	Focal mononuclear infiltration in the heart
	2	Grey-coloured adipose tissue	No histopathological alteration of the adipose tissue, focal interstitial nephritis in the left and right kidney
	3	None	No histopathological alterations
	4	None	No histopathological alterations
	5	None	No histopathological alterations
	6	None	No histopathological alterations
	7	None	Lymphoepithelioid granuloma in the small intestine, focal mononuclear infiltration in the heart
	8	None	Tubular dilation and atrophy in the left and right kidney
	9	None	Cysts in the pituitary gland
	10	None	No histopathological alterations
	11	None	Cortical nodular hyperplasia in the left adrenal gland
	12	None	Cortical nodular hyperplasia in the right adrenal gland, follicular cystic hyperplasia in the thyroid gland and lymphatic sinus ectasia
	13	None	Focal cortical vacuolar degeneration in the right adrenal gland
	14	None	No histopathological alterations
	15	None	No histopathological alterations
	16	None	Focal mononuclear infiltration in the heart
	17	None	No histopathological alterations
	18	None	No histopathological alterations
	19	None	No histopathological alterations
	20	None	Tubular dilation and atrophy in the left and right kidney

**Table 10:** Gross necropsy observations and histopathological findings in female Wistar Han RCC rats fed the control or 33 % GMO diet for 1 year

Group	Animal no.	Gross necropsy observations	Histopathological findings
Control	141	None	No histopathological alterations
	142	None	No histopathological alterations
	143	Cystic formation in the right ovary	Follicular cysts in the right ovary
	144	Bilateral petechial haemorrhage (site: submandibular lymph node), hyperaemic pituitary gland, maxilla deformation, thickened skin area	Cutaneous papilloma, cystic endometrial hyperplasia
	145	Cystic formation in the right ovary	Follicular cysts in the right ovary
	146	Cystic formation in the left and right ovary	Follicular cysts in the left and right ovary
	147	Bilateral hyperaemic submandibular lymph node	Cysts in the thymus, no further histopathological alterations
	148	None	Follicular cysts in the left ovary
	149	None	Tubular dilation and atrophy in the right kidney
	150	None	No histopathological alterations
	151	None	Focal interstitial nephritis in the rat kidney
	152	None	No histopathological alterations
	153	Hyperaemic right submandibular lymph node, petechiae on the left submandibular lymph node, cystic formation in the left and right ovary	Tubular dilation and atrophy in the right kidney, no further histopathological alterations
	154	Bilateral petechial haemorrhage (submandibular lymph node)	No histopathological alterations
	155	None	No histopathological alterations
	156	Diaphragmatic hernia	Diaphragm sample not analysed
	157	Light brown-coloured mass of adipose tissue on the right side of the abdominal cavity	Lipoma
	158	Thickened right horn of the uterus	Cystic endometrial hyperplasia, focal hyperplasia/zona fasciculata in the right adrenal gland
	159	Mammary gland abscess, bilateral petechial haemorrhage (submandibular lymph node)	Mammary gland comedocarcinoma
	160	None	No histopathological alterations

Group	Animal no.	Gross necropsy observations	Histopathological findings
33 % GMO	101	Cystic formation in the right ovary	Follicular cysts in the right ovary
	102	Light brown coloured mass in the epigastrium	Lipoma
	103	Bilateral petechial haemorrhage (site: submandibular lymph node), atrophy of the left and right ovary	Lack of secondary and tertiary follicles in the left and right ovary; no further histopathological alterations
	104	Hyperaemic, dark coloured submandibular lymph node	No histopathological alterations
	105	Hyperaemic pituitary gland	No histopathological alterations
	106	Cystic formation in the left and right ovary	Follicular cysts in the left and right ovary
	107	Superficially damaged skin surface	Skin sample not analysed
	108	None	No histopathological alterations
	109	Cystic formation in the left and right ovary	Follicular cysts in the left and right ovary
	110	Bilateral hyperaemia in the submandibular lymph node, cystic formation in the left and right ovary	Follicular cysts in the left and right ovary
	111	Bilateral hyperaemia in the submandibular lymph node	Endometrial stromal hyperplasia; no further no histopathological alterations
	112	Damaged skin surface	Focal hyperkeratosis, spongiosis, acanthosis, granulation tissue, hypertrophy of the sebaceous glands and follicular dysplasia (site: skin); follicular cysts in the rat ovary
	113	Bilateral hyperaemia in the submandibular lymph node	No histopathological alterations
	114	Bilateral hyperaemia in the submandibular lymph node	No histopathological alterations
	115	None	Lymphoepithelioid granuloma in the small intestine
	116	Bilateral petechial haemorrhage (site: submandibular lymph node)	Tubular dilation and atrophy in the left kidney, no further histopathological alterations
	117	Cystic formation in the left ovary	Follicular cysts in the left ovary, lymphoepithelioid granuloma in the small intestine, cysts in the pituitary gland
	118	Bilateral petechial haemorrhage (site: submandibular lymph node)	No histopathological alterations
	119	None	No histopathological alterations
	120	None	No histopathological alterations

The incidence of the different non-cancerous lesions in the male rats fed the control and the 33 % GMO diets was similar and very low (i.e. in most cases, findings in 1–2 animals per group, if at all, were observed) (Table 11). The incidence of the different non-cancerous lesions in the female rats fed the control and the 33 % GMO diets was similar and very low (i.e. in most cases, findings in 1–2 animals per group were detected), except for cysts in the ovaries, which were observed in 4 out of 19 rats fed the control diet and in 6 out of 20 rats fed the 33 % GMO diet (Table 12). The number of benign and malignant tumours detected in rats fed the control or 33 % GMO diet was extremely low (Table 13). Benign tumours were not observed in male rats of either group, while one out of 19 female rats fed the control diet developed a lipoma, one out of 19 female rats fed the control diet a cutaneous papilloma and one out of 20 female rats fed the 33 % GMO diet a lipoma. Malignant tumours were not detected in male rats of either group, while one out of 20 female rats fed the control diet developed a mammary gland comedocarcinoma. Moreover, a pituitary haemangioma in the conventional 2 group (Table 8, Electronic Supplementary Material) and a yolk sac carcinoma in the 11 % GMO group (Table 9, Electronic Supplementary Material) were observed.

**Table 11:** Summary of the histopathological findings (except for benign and malignant tumours) in male Wistar Han RCC rats fed the control or 33 % GMO diet for 1 year

Organ	Histological finding	Control	33 % GMO
Adrenal gland	Focal hyperplasia/zona fasciculata	0/20	2/19
	Focal cortical vacuolar degeneration	0/20	1/19
Heart	Mononuclear infiltration	3/20	2/19
Hypophysis	Cysts	1/20	1/19
Kidney	Focal interstitial nephritis	1/20	1/19
	Tubular dilation and atrophy	1/20	2/19
Lymph node	Lymphatic sinus ectasia	1/20	1/19
Meibomian gland	Hyperplasia	1/20	0/19
Prostate	Focal chronic interstitial prostatitis	1/20	0/19
Small intestine	Lymphoepithelioid granuloma	1/20	1/19
Thyroid gland	Follicular cystic hyperplasia	0/20	1/19

**Table 12:** Summary of the histopathological findings (except for benign and malignant tumours) in female Wistar Han RCC rats fed the control or 33 % GMO diet for 1 year

Organ	Histological finding	Control	33 % GMO
Adrenal gland	Focal proliferation of interstitial cells	0/19	0/20
Endometrium	Cystic endometrial hyperplasia	2/19	1/20
	Endometrial stromal hyperplasia	0/19	0/20
Hypophysis	Cysts	0/19	1/20
Kidney	Focal interstitial nephritis	1/19	0/20
	Tubular dilation and atrophy	2/19	1/20
Ovary	Cysts	4/19	6/20
	Proliferation of stromal interstitial cells	0/19	0/20
Skin	Focal hyperkeratosis, spongiosis, acanthosis, granulation tissue, hypertrophy of the sebaceous glands and follicular dysplasia	0/19	1/20
Small intestine	Lymphoepithelioid granuloma	0/19	2/20
Thymus	Cysts	1/19	0/20

**Table 13:** Benign and malignant tumours observed in rats fed the control or 33 % GMO diet in the present study

Female rats	Control	33 % GMO
Benign tumours		
Lipoma	1/19	1/20
Cutaneous papilloma	1/19	0/20
Malignant tumours		
Mammary gland comedocarcinoma	1/20	0/20

## Comparison of the SES analysis approach versus the classical statistical analysis approach

The significances identified by SES analysis and those identified by the appropriate classical statistical tests, which were selected according to a decision tree described by the OECD (2012) as well as by Schmidt et al. (2015b), were 99 % in agreement. In the case of 609 endpoint comparisons, the SES approach detected 21 significant differences, whereby four of them were not identified by the classical approach. The classical decision tree-based approach found 19 significant differences, and two of them were not identified by the SES approach.

If solely the Kruskal–Wallis test followed by the Wilcoxon test was applied to all 609 endpoint comparisons, 11 significant differences were detected. If solely the ANOVA followed by the Dunnett test was applied to all 609 endpoint comparisons, 12 significant differences were observed. If solely the ANOVA followed by the *t* test was applied to all 609 endpoint comparisons, 39 significant differences were detected.

## Discussion

### The compositional analysis of the diets

The compositional analysis of the diets showed that the differences between the diets containing the near-isogenic non-GM maize, MON810 maize or conventional 2 maize variety were irrelevant, except for fumonisins in the near-isogenic non-GM maize, and not considered to impair the health of the experimental animals.

**Fig. 7:**
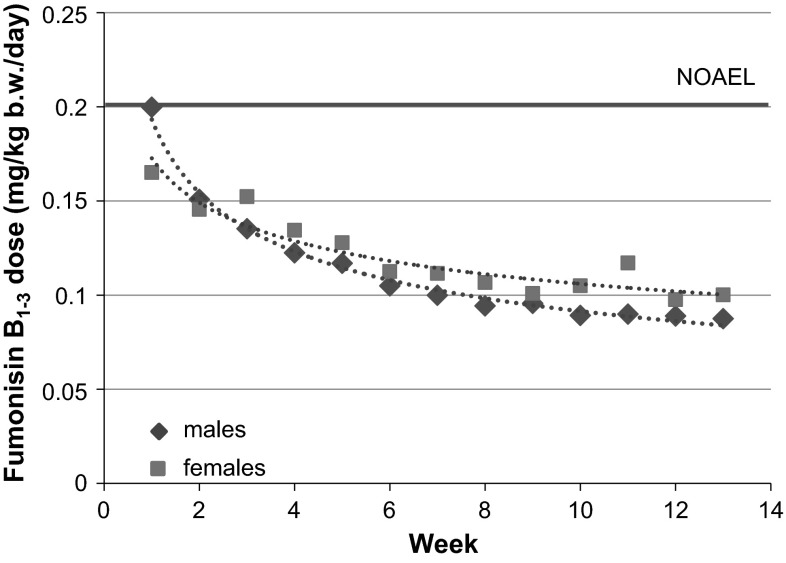
Calculated dose of fumonisins in rats fed the control diet in the first 13 weeks of the 1-year feeding trial. For the calculations, the amount of the fumonisins B_1_, B_2_ and B_3_ were added up. The *red line* represents the lowest no observed adverse effect level (NOAEL) value reported for fumonisin B_1_ in rats, i.e. an NOAEL value of 0.2 mg/kg body weight/day for kidney toxicity in a subchronic toxicity study (Voss et al. 1995)

In order to evaluate whether the contamination of the control diet with fumonisins could have influenced the outcome of the study, the dose of the ingested fumonisins throughout the feeding trial was calculated by taking into account the feed consumption and the body weight. In the scientific literature, the lowest no observed adverse effect level (NOAEL) values reported for fumonisin B_1_ in rats were 0.2 mg/kg body weight/day (endpoint: kidney toxicity) in a subchronic toxicity study (Voss et al. 1995) and 0.25 mg/kg body weight/day (endpoint: kidney toxicity) in a chronic toxicity study (US-NTP 1999). The calculated fumonisin B_1–3_ dose in male rats in the first week of the feeding trial was 0.2 mg/kg body weight/day and thereafter continuously declined until the end of the study, while in female rats the fumonisin B_1–3_ dose was below the value of 0.2 mg/kg body weight/day in the first week of the feeding trial and continued to decrease until the end of the study (Fig. 7 shows the calculated fumonisin B_1–3_ dose for male and female rats during the first 13 weeks of the feeding trial). Hence, at no time during the 1-year feeding trial did the fumonisin B_1–3_ dose in male and female rats exceed the NOAEL value for fumonisin B_1_.

High levels of fumonisins in the diet are known to induce nephrotoxicity and hepatotoxicity in rats (US-NTP 1999). The functional renal and hepatic parameters measured in the blood of the rats fed the control diet for 1 year did not reveal any sign of nephrotoxicity and hepatotoxicity. Males and females of the control group showed no relevant differences in the incidence of histopathological alterations in the kidney if compared to those of the 33 % GMO group, which in both cases was very low, and no preneoplastic and/or neoplastic lesions were identified. No histopathological alterations in the liver of male and female Wistar Han RCC rats fed the control diet for 1 year were observed. Taken together, based on the above-mentioned calculations and determined parameters, it is concluded that the fumonisin contamination of the control diet did not influence the outcome of the study.

MON810 was present in the control and conventional maize varieties at the limit of quantitation (approximately 0.1 %), while in the corresponding diets it was non-detectable in some diet samples and non-quantifiable (i.e. below the limit of quantification) in others. This indicates that MON810 occurred at trace levels at best, various orders of magnitude below that in the maize and diet materials designated as MON810. The source of the admixture was not identified. Given that ≥99.9 % of maize used in the diets fed to the non-GM groups is not MON810, these traces of admixture were not considered to impact on the validity of the comparison of animal groups fed the non-GM diets with those fed MON810 for possible effects linked to MON810 consumption.

### Differences in the mean body weight between control and GMO-fed rats

Although not statistically significant, the male rats fed the 33 % GMO diets had a lower mean body weight than the male animals fed the control diet throughout the study, while there were no differences in the body weight between male rats fed the 11 % GMO diet and those fed the control diet. The mean body weight of female rats fed the 11 % GMO and 33 % GMO diets was lower than that of the animals fed the control diet throughout the study, but the differences were not statistically significant, and the mean body weight of the rats fed the 11 % GMO and 33 % GMO diets was similar. The lower mean body weight of the GMO-fed animals correlated with a lower feed consumption. It was hypothesized that the lower feed intake could be due to an altered hardness and/or particle size distribution of the pellets containing the GM maize. However, an analysis of the physical properties of the different pellets by the Research Institute of Feed Technology (Braunschweig, Germany) revealed no differences regarding their hardness and particle size distribution (data not shown). In the absence of any significant differences in the blood parameters, any significant differences in the relative organ weights and any histopathological alterations that could explain the body weight differences between the groups, it is concluded that the statistically not significant differences in body weight between the experimental groups are of no toxicological concern and are not event-related.

In this context, it is important to point out that in one of the 90-day feeding trials with the GM maize MON810, the so-called study A (Zeljenková et al. 2014) performed in the frame of the GRACE project, a similar body weight decrease was observed in male and female rats fed the conventional variety SY-NEPAL (conventional 2), which was also tested in the present study. As shown in Table 14, the mean body weights of male and female rats fed the conventional 2 diet for 90 days in the study A were about 4–4.5 % lower than those of the corresponding control diet-fed rats. Since study A was finalized after 90 days, a further comparison at later points in time is not possible. Thus, mean body weight variations such as those reported in the present study can also be achieved with conventional maize and without accompanying physiopathological alterations in the animals.

**Table 14:** Comparison of the mean body weight variation in the case of male and female rats fed the control, conventional 2, 11 % GMO or 33 % GMO diet for 90 days in the GRACE project study A (Zeljenková et al. 2014) and in the present study

	Conventional 2 versus control (% mean body weight variation)	11 % GMO versus control (% mean body weight variation)	33& GMO versus control (% mean body weight variation)
Study A			
Males	−4.52	−0.98	−0.50
Females	−3.93	−3.38	−3.92
Present study			
Males	−0.79	1.38	−5.56
Females	−1.54	−3.26	−4.65

### Comparison of the haematology and clinical biochemistry parameters as well as the relative organ weights in control and GMO-fed rats

The following discussion on differences in the haematology and clinical biochemistry parameters as well as in the relative organ weights is based on the differences identified by the SES analyses. After 12 months, the percentage of eosinophils was significantly increased in male rats fed the conventional 2 and 11 % GMO diets, but not in male animals fed the 33 % GMO diet when compared to the control group. At the same sampling time, the percentage of eosinophils was significantly decreased in female rats fed the conventional 2 and 11 % GMO diets, while in female rats fed the 33 % GMO diet the percentage of eosinophils significantly increased after 3 months and significantly decreased after 6 months, while it was not statistically different from that in the control group after 12 months. The number of WBC was significantly increased in male rats fed the 11 and 33 % GMO diet after 6 months when compared to control diet-fed animals. No differences were detected in male rats either after 3 and 12 months or in female animals throughout the whole feeding trial. Taken together, there is no evidence of an event-related effect of the GM maize MON810 on the percentage of eosinophils in the blood, which is in line with the observation that (Cry1Ab-containing) maize MON810 does not induce relevant changes in immune parameters when rats are fed a 33 % GMO diet for up to 90 days (Tulinska et al., manuscript in preparation), and on the number of WBC.

In the case of the clinical biochemistry data, a low number of parameters were significantly altered when the control and the GMO groups were compared. After publishing the results of the first two 90-day feeding trials, studies A and B, in the frame of the GRACE project (Zeljenková et al. 2014), it was argued in the frame of discussions with the stakeholders that: (1) the GRACE consortium had dismissed the toxicological relevance of the observed lower levels of TP in serum in rats fed the GM maize MON810, which could be due to a nephrotic syndrome or to an impaired protein synthesis in the liver; (2) the GRACE consortium had dismissed the decrease in the relative pancreas weight and the increase in the blood GLU levels. The results of the present study show that: (1) the blood TP levels remained unchanged in male and female rats fed the 11 % GMO and 33 % GMO diets for up to 1 year when compared to the control group; (2) the histopathological analyses of the kidneys and liver of the rats fed the 33 % GMO diet revealed no signs of a nephrotic syndrome or liver toxicity; (3) the relative pancreas weight remained unchanged in male and female rats fed the 11 % GMO and 33 % GMO diets for up to 1 year when compared to the control group; and (4) the blood GLU levels in male and female rats fed the 11 % GMO and 33 % GMO diets for up to 1 year in no case increased when compared to the control group. Hence, the conclusions raised in the study by Zeljenková et al. (2014) and commented by Steinberg (2015) are clearly supported and confirmed by the results obtained in the present 1-year feeding trial with the GM maize MON810.

Blood AST activity was only decreased in male rats fed the conventional 2 diet after 3 months and increased in male rats fed the 33 % GMO diet for 6 months, while other liver function-related biomarkers such as blood ALP and ALT activities as well as blood TP and ALB levels remained unchanged in those animals when compared to the corresponding control group. No liver function-related biomarkers were altered in male rats fed the GMO diets for 3 and 12 months and in female rats fed the GMO diets for 3, 6 and 12 months. Moreover, no histopathological alterations were observed in male and female rats fed the GMO diets. Thus, it is concluded that the GM maize feeding did not lead to liver toxicity and that the above-mentioned AST activity increase was not event-related.

The blood GLU level in female rats fed the 33 % GMO was significantly lower after 6 months if compared to the corresponding control animals, but this was not the case after 12 months. The blood GLU levels in male rats fed the 11 % GMO diet were significantly lower after 12 months, while the blood GLU level in male animals fed the 33 % GMO diet for 12 months showed no alterations. Based on the above-mentioned results, it is concluded that the sporadic decrease in blood glucose levels observed in the 1-year feeding trial with the GM maize MON810 was not related to the MON810 event.

The blood CREA level was slightly increased in female rats fed the 11 % GMO diet for 12 months, but not in female rats fed the 33 % GMO diet for 12 months or in male rats fed the 11 % GMO and 33 % GMO diets for up to 12 months. In this context, it should be pointed out that the increase in the blood CREA level in female rats fed the 11 % GMO diet for 12 months was not accompanied by changes in further kidney-related parameters such as blood U and electrolyte levels. Moreover, there was no relevant increase in the incidence of histopathological alterations in the kidneys of animals fed the 33 % GMO diet if compared with the control group. Therefore, it is concluded that the feeding of maize MON810 did not lead to kidney toxicity and that the above-mentioned CREA increase was not event-related.

The blood P levels were increased in male rats fed the 11 % GMO and 33 % GMO diets to a similar extent (i.e. in a dose-independent manner) after 6 months and slightly increased in male rats fed the 11 % GMO (but not the 33 % GMO) diet after 12 months when compared to the corresponding control group. The blood Cl level was increased in male rats fed the 33 % GMO diet after 3 months, whereas after 6 and 12 months no changes in the blood Cl levels of male rats were detected. In female rats, the blood P and Cl levels were not altered at any point in time of the feeding trial. If one takes into account that the above-mentioned changes in the blood P and Cl levels were not accompanied by changes in kidney-related parameters such as blood U, CREA and further electrolyte levels and that there was no relevant increase in the incidence of histopathological alterations in the kidneys of animals fed the 33 % GMO diet when compared with the control group, it is concluded that the feeding of maize MON810 did not lead to kidney toxicity and that the P and Cl increases were not event-related.

The only additional significantly altered clinical biochemistry parameter detected by the classical statistical analysis approach was an increase in the AST activity in female rats fed the 33 % GMO diet for 12 months. Since all other liver function-related biomarkers such as blood ALP and ALT activities as well as blood TP and ALB levels remained unchanged in female animals when compared to the corresponding control group, no liver function-related biomarkers were altered in male rats fed the GMO diets for 3, 6 and 12 months and no histopathological alterations were observed in male and female rats fed the 33 % GMO diet, it is concluded that the GM maize feeding did not lead to liver toxicity and that the above-mentioned AST activity increase in female rats was not event-related.

### Necropsy and histopathological findings

The macroscopic examination of all animals at necropsy revealed a very limited number of findings in all four experimental groups. The histopathological analysis of the organs and tissues obtained from all animals of the control and 33 % GMO group identified a very low number of alterations in both groups. Since there were no relevant differences in the incidence of histopathological findings between these two groups, it is concluded that the alterations were not event-related.

The very low incidence of non-neoplastic and neoplastic lesions in Wistar Han RCC rats is in line with previous reports (King-Herbert and Thayer 2006; Weber et al. 2011). Thus, these findings support the use of Wistar Han RCC rats in chronic toxicity and carcinogenicity studies. One conventional 2-fed female rat developed a pituitary haemangioma and one 11 % GMO-fed female rat a yolk sac carcinoma, a rare spontaneous neoplasm of germ cell origin (Sobis 1987; Nakazawa et al. 1998; Sakamoto et al. 2011). No malignant tumours were detected in 33 % GMO-fed rats.

### Statistical methods: SES analysis versus classical statistical analysis

In the present study paper, the endpoints measured in the different experimental groups were compared applying both the SES analysis as recommended by EFSA (EFSA Scientific Committee 2011) and the classical statistical approach, i.e. decision tree-based, as recommended by the OECD (OECD 2012). The classical approach (significance tests) involved the comparison of the group means for each endpoint, i.e. difference tests to identify statistically significant group differences (=effect sizes). The SES approach estimates are displayed as confidence intervals for the (standardized) differences of the means.

The results of the SES approach very closely (by 99 %) agree with those of the classical statistical approach, which simply asks whether there is an effect. The confidence intervals estimated by the SES approach additionally provide information on the magnitude of any response, i.e. how strong the effect is.

In the frame of the present study, all endpoints were also analysed by applying a single classical statistical test procedure, a one-way ANOVA followed by post hoc *t* test, a one-way ANOVA followed by post hoc Dunnett test and the Kruskal–Wallis test followed by the Wilcoxon test. The one-way ANOVA followed by post hoc *t* test detected the highest total number of significant differences, i.e. about twice as much as the decision tree-based classical approach (39 vs. 19). The ANOVA followed by post hoc Dunnett test found a low total number of significant differences, i.e. about half as much as the decision tree-based classical approach (12 vs. 19). The Kruskal–Wallis test followed by the Wilcoxon test found the lowest total number of significant differences, i.e. about half as much as the decision tree-based classical approach (11 vs. 19). This is due to:

- the (non-)appropriateness of an applied statistical method to an endpoint (the assumptions underlying the specific test are (not) met);
- the different philosophies behind the statistical methods: ANOVA with post hoc *t* test /Dunnett test is based on the original values, whereas the nonparametric Kruskal–Wallis test followed by the Wilcoxon test is not based on the original values itself, but on their ranks;
- the different approaches to control the probability of type I errors (in contrast to the *t* test, the Dunnett test controls the experimentwise error).

Taken together, the *t* test is a very conservative and cautious approach that detects a rather high number of significant differences, while the nonparametric test is an approach that detects a rather low number of significant differences. In contrast, the decision tree-based classical statistical approach and the SES approach provide a balanced and reliable analysis of the results.

At the present time, the SES approach is unfamiliar to most toxicologists. The standardization allows to compare all endpoints at a glance and helps in evaluating the toxicological relevance of the results obtained. In this context, the use of the SES approach provides an added value to the classical statistical analyses applied in feeding trials with whole food/feed, as shown in the present study.

The advantages of (standardized) effect size estimations have comprehensively been described and discussed by Ellis (2010), Festing (2014) and Schmidt et al. (2015b) and are summarized as follows:

- Assessing the magnitude of an effect instead of just its statistical significance supports the interpretation of its toxicological relevance.
- References to historical data are easier to interpret having the variation of effect sizes displayed in form of confidence intervals.
- For significance tests, graphical methods are not widely available, while for effect sizes a range of graphical methods are applicable.
- By making use of standardization, all endpoints of a feeding trial can be expressed in the same units and displayed in a single graph.

In conclusion, the 1-year feeding trial performed with a MON810 maize variety in the frame of the GRACE project shows that the MON810 maize at a level of up to 33 % in the diet does not lead to toxicologically relevant effects in male and female Wistar Han RCC rats after a 1-year exposure, independently of the applied statistical analysis approach.

### Suitability of rat feeding studies for the health risk assessment of GM food and feed

There is an ongoing discussion in the EU whether untargeted feeding trials in rodents with whole GM food/feed should become a mandatory testing procedure to investigate any unanticipated effect of GM food/feed in the frame of the risk assessment process. In this context, one of the main objectives of the GRACE project was to test GM maize MON810 varieties in subchronic (Zeljenková et al. 2014; Schmidt et al. 2016) and chronic rat feeding trials (the present study) and, based on the obtained results, to evaluate whether such feeding trials are suited to reveal such unintended effects elicited by the genetic modification by an “untargeted” testing, i.e. without a triggering assumption resulting from preceding investigations on a potential effect and, hence, without a specified cause-effect hypothesis. The data did not provide any indication that the performance of the rat feeding studies with whole food/feed would provide additional information on the safety of the GM maize MON810 when compared to the compositional comparison of the GM line and its closest conventional comparator. Moreover, the data generated by the GRACE project showed that non-targeted feeding studies may generate outcomes at the level of the variability of the laboratory performing the studies, i.e. generating significant differences in a random manner, and suggest that animal feeding trials may lack the sensitivity needed to detect unintended effects elicited by the genetic modification.

The need to provide nutritionally balanced diets limits the maximum inclusion rate of the plant material to be tested and, thus, restricts the exposure level of the animals to the respective GM food/feed. In this context, the expected magnitude of a distinctly identified potential effect should be included into the test hypothesis and trigger the decision whether a feeding study should/could be performed to achieve a clear test response. The study design should follow the test hypothesis and should provide clear test results. A standard approach may not be the primary choice to achieve this objective.

Taken together, the GRACE data support the scientific reasoning that only in case that a trigger has been identified in the course of the initial molecular, compositional, phenotypic and/or agronomic analyses, feeding trials with whole food/feed may provide an added scientific value for the risk assessment of GM crops (EFSA Scientific Committee 2011). If safety concerns are raised during the molecular, compositional, phenotypic and/or agronomic analyses, a feeding trial might be considered, provided that a targeted hypothesis can be developed to tailor the study design to the posed safety concern.

In line with the GRACE transparency policy, any interested person will have access to the raw data obtained in the frame of GRACE, including the clinical, ophthalmological, haematology, clinical biochemistry, organ weight, necropsy and histopathology data presented in this study through an internet portal named CADIMA (Central Access Database for Impact Assessment of Crop Genetic Improvement Technologies; www.cadima.info).

## Electronic supplementary material

Below is the link to the electronic supplementary material.

Supplementary material 1 (DOCX 36 kb)

Supplementary material 2 (DOCX 33 kb)

Supplementary material 3 (PDF 103 kb)

Supplementary material 4 (XLSX 23 kb)

Supplementary material 5 (XLSX 24 kb)

Supplementary material 6 (DOCX 21 kb)

Supplementary material 7 (DOCX 20 kb)

Supplementary material 8 (DOCX 21 kb)

Supplementary material 9 (DOCX 19 kb)

Supplementary material 10 (DOCX 19 kb)

## Footnotes

1 The “conventional 2” group is named like that, since it refers to the same “conventional 2” variety (SY-NEPAL) tested in the 90-day feeding trial A previously described by Zeljenková et al. (2014).

2 The body weight and feed consumption data were analysed by applying mixed models. In this context, a statistically significant difference means that the mixed model analysis identified a group effect.

3 For all the haematology and clinical biochemistry endpoints as well as for the relative organ weights, SES was calculated. In this context, a statistically significant difference means that the confidence interval of the SES to the control does not include the zero value, while a difference, which is not statistically significant, means that the confidence interval of the SES to the control includes the zero value (Fig. 1).

4 Slides of all tissues analysed may be looked at on request at the Laboratory of Toxicology of the Slovak Medical University (Bratislava, Slovakia).
