# Supplementary material for: One-year oral toxicity study on a genetically modified maize MON810 variety in Wistar Han RCC rats (EU 7th Framework Programme project GRACE)
Source: Arch Toxicol. 2016 Jul 20;90(10):2531–62. doi: 10.1007/s00204-016-1798-4 (PMC5043003; doi:10.1007/s00204-016-1798-4)
Supplement: Supplementary file 1 — Supplementary material 1 (DOCX 36 kb) [file 204_2016_1798_MOESM1_ESM.docx]

**ESM-Fig. 1:** Meteorological data in 2013, compared to the mean data 1984-2012
