# Supplementary material for: One-year oral toxicity study on a genetically modified maize MON810 variety in Wistar Han RCC rats (EU 7th Framework Programme project GRACE)
Source: Arch Toxicol. 2016 Jul 20;90(10):2531–62. doi: 10.1007/s00204-016-1798-4 (PMC5043003; doi:10.1007/s00204-016-1798-4)
Supplement: Supplementary file 3 — Supplementary material 3 (PDF 103 kb) [file 204_2016_1798_MOESM3_ESM.pdf]

ESM-Table 2: Sampling order at the different points in time of the 1-year feeding trial

3 months

Cage_ID	Anim_ID	Group_ID	Sex	Date	Time
1	1	33% GMO	M	28.4.2014	7:00-10:00
	2	33% GMO	M	28.4.2014	7:00-10:00
4	7	33% GMO	M	28.4.2014	7:00-10:00
	8	33% GMO	M	28.4.2014	7:00-10:00
12	23	11% GMO	M	28.4.2014	7:00-10:00
	24	11% GMO	M	28.4.2014	7:00-10:00
13	25	11% GMO	M	28.4.2014	7:00-10:00
	26	11% GMO	M	28.4.2014	7:00-10:00
21	41	control	M	28.4.2014	7:00-10:00
	42	control	M	28.4.2014	7:00-10:00
22	43	control	M	28.4.2014	7:00-10:00
	44	control	M	28.4.2014	7:00-10:00
31	61	conventional 2	M	28.4.2014	7:00-10:00
	62	conventional 2	M	28.4.2014	7:00-10:00
34	67	conventional 2	M	28.4.2014	7:00-10:00
	68	conventional 2	M	28.4.2014	7:00-10:00
5	9	33% GMO	M	29.4.2014	7:00-9:40
	10	33% GMO	M	29.4.2014	7:00-9:40
9	17	33% GMO	M	29.4.2014	7:00-9:40
	18	33% GMO	M	29.4.2014	7:00-9:40
15	29	11% GMO	M	29.4.2014	7:00-9:40
	30	11% GMO	M	29.4.2014	7:00-9:40
17	33	11% GMO	M	29.4.2014	7:00-9:40
	34	11% GMO	M	29.4.2014	7:00-9:40
25	49	control	M	29.4.2014	7:00-9:40
	50	control	M	29.4.2014	7:00-9:40
26	51	control	M	29.4.2014	7:00-9:40
	52	control	M	29.4.2014	7:00-9:40
36	71	conventional 2	M	29.4.2014	7:00-9:40
	72	conventional 2	M	29.4.2014	7:00-9:40
38	75	conventional 2	M	29.4.2014	7:00-9:40
	76	conventional 2	M	29.4.2014	7:00-9:40
10	19	33% GMO	M	30.4.2014	7:00-9:50
	20	33% GMO	M	30.4.2014	7:00-9:50
51	101	33% GMO	F	30.4.2014	7:00-9:50
	102	33% GMO	F	30.4.2014	7:00-9:50
19	37	11% GMO	M	30.4.2014	7:00-9:50
	38	11% GMO	M	30.4.2014	7:00-9:50
62	123	11% GMO	F	30.4.2014	7:00-9:50
	124	11% GMO	F	30.4.2014	7:00-9:50
28	55	control	M	30.4.2014	7:00-9:50
	56	control	M	30.4.2014	7:00-9:50
73	145	control	F	30.4.2014	7:00-9:50

Sampling order

[illegible]	146	control	F	30.4.2014	7:00-9:50
39	77	conventional 2	M	30.4.2014	7:00-9:50
	78	conventional 2	M	30.4.2014	7:00-9:50
81	161	conventional 2	F	30.4.2014	7:00-9:50
	162	conventional 2	F	30.4.2014	7:00-9:50
52	103	33% GMO	F	1.5.2014	7:00-10:00
	104	33% GMO	F	1.5.2014	7:00-10:00
54	107	33% GMO	F	1.5.2014	7:00-10:00
	108	33% GMO	F	1.5.2014	7:00-10:00
64	127	11% GMO	F	1.5.2014	7:00-10:00
	128	11% GMO	F	1.5.2014	7:00-10:00
68	135	11% GMO	F	1.5.2014	7:00-10:00
	136	11% GMO	F	1.5.2014	7:00-10:00
74	147	control	F	1.5.2014	7:00-10:00
	148	control	F	1.5.2014	7:00-10:00
76	151	control	F	1.5.2014	7:00-10:00
	152	control	F	1.5.2014	7:00-10:00
83	165	conventional 2	F	1.5.2014	7:00-10:00
	166	conventional 2	F	1.5.2014	7:00-10:00
85	169	conventional 2	F	1.5.2014	7:00-10:00
	170	conventional 2	F	1.5.2014	7:00-10:00
55	109	33% GMO	F	2.5.2014	7:00- 8:35
	110	33% GMO	F	2.5.2014	7:00- 8:35
57	113	33% GMO	F	2.5.2014	7:00- 8:35
	114	33% GMO	F	2.5.2014	7:00- 8:35
69	137	11% GMO	F	2.5.2014	7:00- 8:35
	138	11% GMO	F	2.5.2014	7:00- 8:35
70	139	11% GMO	F	2.5.2014	7:00- 8:35
	140	11% GMO	F	2.5.2014	7:00- 8:35
79	157	control	F	2.5.2014	7:00- 8:35
	158	control	F	2.5.2014	7:00- 8:35
80	159	control	F	2.5.2014	7:00- 8:35
	160	control	F	2.5.2014	7:00- 8:35
88	175	conventional 2	F	2.5.2014	7:00- 8:35
	176	conventional 2	F	2.5.2014	7:00- 8:35
89	177	conventional 2	F	2.5.2014	7:00- 8:35
	178	conventional 2	F	2.5.2014	7:00- 8:35

6 months

Cage_ID	Anim_ID	Group_ID	Sex	Date	Time
1	1	33% GMO	M	21.07.2014	7:00-9:20
	2	33% GMO	M	21.07.2014	7:00-9:20
4	7	33% GMO	M	21.07.2014	7:00-9:20
	8	33% GMO	M	21.07.2014	7:00-9:20
12	23	11% GMO	M	21.07.2014	7:00-9:20
	24	11% GMO	M	21.07.2014	7:00-9:20
13	25	11% GMO	M	21.07.2014	7:00-9:20
	26	11% GMO	M	21.07.2014	7:00-9:20
21	41	control	M	21.07.2014	7:00-9:20
	42	control	M	21.07.2014	7:00-9:20
22	43	control	M	21.07.2014	7:00-9:20
	44	control	M	21.07.2014	7:00-9:20
31	61	conventional 2	M	21.07.2014	7:00-9:20
	62	conventional 2	M	21.07.2014	7:00-9:20
34	67	conventional 2	M	21.07.2014	7:00-9:20
	68	conventional 2	M	21.07.2014	7:00-9:20
5	9	33% GMO	M	22.07.2014	7:00-9:00
	10	33% GMO	M	22.07.2014	7:00-9:00
9	17	33% GMO	M	22.07.2014	7:00-9:00
	18	33% GMO	M	22.07.2014	7:00-9:00
15	29	11% GMO	M	22.07.2014	7:00-9:00
	30	11% GMO	M	22.07.2014	7:00-9:00
17	33	11% GMO	M	22.07.2014	7:00-9:00
	34	11% GMO	M	22.07.2014	7:00-9:00
25	49	control	M	22.07.2014	7:00-9:00
	50	control	M	22.07.2014	7:00-9:00
26	51	control	M	22.07.2014	7:00-9:00
	52	control	M	22.07.2014	7:00-9:00
36	71	conventional 2	M	22.07.2014	7:00-9:00
	72	conventional 2	M	22.07.2014	7:00-9:00
38	75	conventional 2	M	22.07.2014	7:00-9:00
	76	conventional 2	M	22.07.2014	7:00-9:00
10	19	33% GMO	M	23.07.2014	7:00-9:00
	20	33% GMO	M	23.07.2014	7:00-9:00
51	101	33% GMO	F	23.07.2014	7:00-9:00
	102	33% GMO	F	23.07.2014	7:00-9:00
19	37	11% GMO	M	23.07.2014	7:00-9:00
	38	11% GMO	M	23.07.2014	7:00-9:00
62	123	11% GMO	F	23.07.2014	7:00-9:00
	124	11% GMO	F	23.07.2014	7:00-9:00
28	55	control	M	23.07.2014	7:00-9:00
	56	control	M	23.07.2014	7:00-9:00
73	145	control	F	23.07.2014	7:00-9:00
	146	control	F	23.07.2014	7:00-9:00
39	77	conventional 2	M	23.07.2014	7:00-9:00

Sampling order

[illegible]	78	conventional 2	M	23.07.2014	7:00-9:00
81	161	conventional 2	F	23.07.2014	7:00-9:00
	162	conventional 2	F	23.07.2014	7:00-9:00
52	103	33% GMO	F	24.07.2014	7:00-9:00
	104	33% GMO	F	24.07.2014	7:00-9:00
54	107	33% GMO	F	24.07.2014	7:00-9:00
	108	33% GMO	F	24.07.2014	7:00-9:00
64	127	11% GMO	F	24.07.2014	7:00-9:00
	128	11% GMO	F	24.07.2014	7:00-9:00
68	135	11% GMO	F	24.07.2014	7:00-9:00
	136	11% GMO	F	24.07.2014	7:00-9:00
74	147	control	F	24.07.2014	7:00-9:00
	148	control	F	24.07.2014	7:00-9:00
76	151	control	F	24.07.2014	7:00-9:00
	152	control	F	24.07.2014	7:00-9:00
83	165	conventional 2	F	24.07.2014	7:00-9:00
	166	conventional 2	F	24.07.2014	7:00-9:00
85	169	conventional 2	F	24.07.2014	7:00-9:00
	170	conventional 2	F	24.07.2014	7:00-9:00
55	109	33% GMO	F	25.07.2014	7:00-10:00
	110	33% GMO	F	25.07.2014	7:00-10:00
57	113	33% GMO	F	25.07.2014	7:00-10:00
	114	33% GMO	F	25.07.2014	7:00-10:00
69	137	11% GMO	F	25.07.2014	7:00-10:00
	138	11% GMO	F	25.07.2014	7:00-10:00
70	139	11% GMO	F	25.07.2014	7:00-10:00
	140	11% GMO	F	25.07.2014	7:00-10:00
79	157	control	F	25.07.2014	7:00-10:00
	158	control	F	25.07.2014	7:00-10:00
80	159	control	F	25.07.2014	7:00-10:00
	160	control	F	25.07.2014	7:00-10:00
88	175	conventional 2	F	25.07.2014	7:00-10:00
	176	conventional 2	F	25.07.2014	7:00-10:00
89	177	conventional 2	F	25.07.2014	7:00-10:00
	178	conventional 2	F	25.07.2014	7:00-10:00

12 months

Cage_ID	Anim_ID	Group_ID	Sex	Date	Time
1	1	33% GMO	M	19.01.2015	7:30-8:00
1	2	33% GMO	M	19.01.2015	7:30-8:00
4	7	33% GMO	M	19.01.2015	7:30-8:00
4	8	33% GMO	M	19.01.2015	7:30-8:00
12	23	11% GMO	M	19.01.2015	7:30-8:00
12	24	11% GMO	M	19.01.2015	7:30-8:00
13	25	11% GMO	M	19.01.2015	7:30-8:00
13	26	11% GMO	M	19.01.2015	7:30-8:00
21	41	control	M	19.01.2015	8:00-8:30
21	42	control	M	19.01.2015	8:00-8:30
22	43	control	M	19.01.2015	8:00-8:30
22	44	control	M	19.01.2015	8:00-8:30
31	61	conventional 2	M	19.01.2015	8:00-8:30
31	62	conventional 2	M	19.01.2015	8:00-8:30
34	67	conventional 2	M	19.01.2015	8:00-8:30
34	68	conventional 2	M	19.01.2015	8:00-8:30
2	3	33% GMO	M	19.01.2015	9:00-9:30
2	4	33% GMO	M	19.01.2015	9:00-9:30
3	5	33% GMO	M	19.01.2015	9:00-9:30
2	6	33% GMO	M	19.01.2015	9:00-9:30
11	21	11% GMO	M	19.01.2015	9:00-9:30
11	22	11% GMO	M	19.01.2015	9:00-9:30
14	27	11% GMO	M	19.01.2015	9:00-9:30
14	28	11% GMO	M	19.01.2015	9:00-9:30
23	45	control	M	19.01.2015	9:30-10:00
23	46	control	M	19.01.2015	9:30-10:00
24	47	control	M	19.01.2015	9:30-10:00
24	48	control	M	19.01.2015	9:30-10:00
32	63	conventional 2	M	19.01.2015	9:30-10:00
32	64	conventional 2	M	19.01.2015	9:30-10:00
33	65	conventional 2	M	19.01.2015	9:30-10:00
33	66	conventional 2	M	19.01.2015	9:30-10:00
5	9	33% GMO	M	20.01.2015	7:30-8:00
5	10	33% GMO	M	20.01.2015	7:30-8:00
9	17	33% GMO	M	20.01.2015	7:30-8:00
9	18	33% GMO	M	20.01.2015	7:30-8:00
15	29	11% GMO	M	20.01.2015	7:30-8:00
15	30	11% GMO	M	20.01.2015	7:30-8:00
17	33	11% GMO	M	20.01.2015	7:30-8:00
17	34	11% GMO	M	20.01.2015	7:30-8:00
25	49	control	M	20.01.2015	8:00-8:30
25	50	control	M	20.01.2015	8:00-8:30
26	51	control	M	20.01.2015	8:00-8:30
26	52	control	M	20.01.2015	8:00-8:30
36	71	conventional 2	M	20.01.2015	8:00-8:30

Sampling order

36	72	conventional 2	M	20.01.2015	8:00-8:30
38	75	conventional 2	M	20.01.2015	8:00-8:30
38	76	conventional 2	M	20.01.2015	8:00-8:30
6	11	33% GMO	M	20.01.2015	9:00-9:30
6	12	33% GMO	M	20.01.2015	9:00-9:30
7	13	33% GMO	M	20.01.2015	9:00-9:30
7	14	33% GMO	M	20.01.2015	9:00-9:30
16	31	11% GMO	M	20.01.2015	9:00-9:30
16	32	11% GMO	M	20.01.2015	9:00-9:30
18	35	11% GMO	M	20.01.2015	9:00-9:30
18	36	11% GMO	M	20.01.2015	9:00-9:30
27	53	control	M	20.01.2015	9:30-10:00
27	54	control	M	20.01.2015	9:30-10:00
29	57	control	M	20.01.2015	9:30-10:00
29	58	control	M	20.01.2015	9:30-10:00
35	69	conventional 2	M	20.01.2015	9:30-10:00
35	70	conventional 2	M	20.01.2015	9:30-10:00
37	73	conventional 2	M	20.01.2015	9:30-10:00
37	74	conventional 2	M	20.01.2015	9:30-10:00
10	19	33% GMO	M	21.01.2015	7:30-8:00
10	20	33% GMO	M	21.01.2015	7:30-8:00
19	37	11% GMO	M	21.01.2015	7:30-8:00
19	38	11% GMO	M	21.01.2015	7:30-8:00
51	101	33% GMO	F	21.01.2015	7:30-8:00
51	102	33% GMO	F	21.01.2015	7:30-8:00
62	123	11% GMO	F	21.01.2015	7:30-8:00
62	124	11% GMO	F	21.01.2015	7:30-8:00
28	55	control	M	21.01.2015	8:00-8:30
28	56	control	M	21.01.2015	8:00-8:30
39	77	conventional 2	M	21.01.2015	8:00-8:30
39	78	conventional 2	M	21.01.2015	8:00-8:30
73	145	control	F	21.01.2015	8:00-8:30
73	146	control	F	21.01.2015	8:00-8:30
81	161	conventional 2	F	21.01.2015	8:00-8:30
81	162	conventional 2	F	21.01.2015	8:00-8:30
8	16	33% GMO	M	21.01.2015	8:30-9:00
20	39	11% GMO	M	21.01.2015	8:30-9:00
20	40	11% GMO	M	21.01.2015	8:30-9:00
60	119	33% GMO	F	21.01.2015	8:30-9:00
60	120	33% GMO	F	21.01.2015	8:30-9:00
67	133	11% GMO	F	21.01.2015	8:30-9:00
67	134	11% GMO	F	21.01.2015	9:00-9:30
30	59	control	M	21.01.2015	9:00-9:30
30	60	control	M	21.01.2015	9:00-9:30
40	79	conventional 2	M	21.01.2015	9:00-9:30
40	80	conventional 2	M	21.01.2015	9:00-9:30
78	155	control	F	21.01.2015	9:00-9:30
78	156	control	F	21.01.2015	9:00-9:30
90	179	conventional 2	F	21.01.2015	9:00-9:30
90	180	conventional 2	F	21.01.2015	9:00-9:30

52	103	33% GMO	F	22.01.2015	7:30-8:00
52	104	33% GMO	F	22.01.2015	7:30-8:00
54	107	33% GMO	F	22.01.2015	7:30-8:00
54	108	33% GMO	F	22.01.2015	7:30-8:00
64	127	11% GMO	F	22.01.2015	7:30-8:00
64	128	11% GMO	F	22.01.2015	7:30-8:00
68	135	11% GMO	F	22.01.2015	8:00-8:30
68	136	11% GMO	F	22.01.2015	8:00-8:30
74	147	control	F	22.01.2015	8:00-8:30
74	148	control	F	22.01.2015	8:00-8:30
76	151	control	F	22.01.2015	8:00-8:30
76	152	control	F	22.01.2015	8:00-8:30
83	165	conventional 2	F	22.01.2015	8:00-8:30
83	166	conventional 2	F	22.01.2015	8:00-8:30
85	169	conventional 2	F	22.01.2015	8:30-9:00
85	170	conventional 2	F	22.01.2015	8:30-9:00
53	105	33% GMO	F	22.01.2015	8:30-9:00
53	106	33% GMO	F	22.01.2015	8:30-9:00
56	111	33% GMO	F	22.01.2015	8:30-9:00
56	112	33% GMO	F	22.01.2015	8:30-9:00
61	121	11% GMO	F	22.01.2015	9:00-9:30
61	122	11% GMO	F	22.01.2015	9:00-9:30
63	125	11% GMO	F	22.01.2015	9:00-9:30
63	126	11% GMO	F	22.01.2015	9:00-9:30
71	141	control	F	22.01.2015	9:00-9:30
71	142	control	F	22.01.2015	9:00-9:30
72	143	control	F	22.01.2015	9:00-9:30
72	144	control	F	22.01.2015	9:00-9:30
82	163	conventional 2	F	22.01.2015	9:30-10:00
82	164	conventional 2	F	22.01.2015	9:30-10:00
84	167	conventional 2	F	22.01.2015	9:30-10:00
84	168	conventional 2	F	22.01.2015	9:30-10:00
55	109	33% GMO	F	23.01.2015	7:15-7:45
55	110	33% GMO	F	23.01.2015	7:15-7:45
57	113	33% GMO	F	23.01.2015	7:15-7:45
57	114	33% GMO	F	23.01.2015	7:15-7:45
69	137	11% GMO	F	23.01.2015	7:15-7:45
69	138	11% GMO	F	23.01.2015	7:15-7:45
70	139	11% GMO	F	23.01.2015	7:15-7:45
70	140	11% GMO	F	23.01.2015	7:15-7:45
79	157	control	F	23.01.2015	7:45-8:30
79	158	control	F	23.01.2015	7:45-8:30
80	160	control	F	23.01.2015	7:45-8:30
88	175	conventional 2	F	23.01.2015	7:45-8:30
88	176	conventional 2	F	23.01.2015	7:45-8:30
89	177	conventional 2	F	23.01.2015	7:45-8:30
89	178	conventional 2	F	23.01.2015	7:45-8:30
58	115	33% GMO	F	23.01.2015	8:30-9:00
58	116	33% GMO	F	23.01.2015	8:30-9:00
59	117	33% GMO	F	23.01.2015	8:30-9:00

59	118	33% GMO	F	23.01.2015	8:30-9:00
65	129	11% GMO	F	23.01.2015	8:30-9:00
65	130	11% GMO	F	23.01.2015	8:30-9:00
66	132	11% GMO	F	23.01.2015	9:00-9:30
75	149	control	F	23.01.2015	9:00-9:30
75	150	control	F	23.01.2015	9:00-9:30
77	153	control	F	23.01.2015	9:00-9:30
77	154	control	F	23.01.2015	9:00-9:30
86	171	conventional 2	F	23.01.2015	9:00-9:30
86	172	conventional 2	F	23.01.2015	9:00-9:30
87	173	conventional 2	F	23.01.2015	9:00-9:30
87	174	conventional 2	F	23.01.2015	9:30-10:00

Necropsy

Cage_ID	Anim_ID	Group_ID	Sex	Blood taking Start-h	Day Necropsy	Date necropsy
16	31	11% GMO	M	09:56	366	27.01.2015
16	32	11% GMO	M	10:20	366	27.01.2015
21	41	control	M	10:44	366	27.01.2015
21	42	control	M	10:59	366	27.01.2015
31	61	conventional 2	M	11:15	366	27.01.2015
31	62	conventional 2	M	11:29	366	27.01.2015
10	19	33% GMO	M	11:53	366	27.01.2015
10	20	33% GMO	M	12:05	366	27.01.2015
28	55	control	M	13:31	366	27.01.2015
28	56	control	M	13:57	366	27.01.2015
40	79	conventional 2	M	14:15	366	27.01.2015
40	80	conventional 2	M	14:25	366	27.01.2015
14	27	11% GMO	M	14:43	366	27.01.2015
14	28	11% GMO	M	14:50	366	27.01.2015
4	7	33% GMO	M	15:05	366	27.01.2015
4	8	33% GMO	M	15:20	366	27.01.2015
65	129	11% GMO	F	07:35	366	28.01.2015
65	130	11% GMO	F	07:43	366	28.01.2015
56	111	33% GMO	F	08:30	366	28.01.2015
56	112	33% GMO	F	08:46	366	28.01.2015
86	171	conventional 2	F	9:05	366	28.01.2015
86	172	conventional 2	F	9:16	366	28.01.2015
76	151	control	F	9:35	366	28.01.2015
76	152	control	F	9:57	366	28.01.2015
64	127	11% GMO	F	12:00	366	28.01.2015
64	128	11% GMO	F	12:15	366	28.01.2015
54	107	33% GMO	F	12:27	366	28.01.2015
54	108	33% GMO	F	12:37	366	28.01.2015
88	175	conventional 2	F	12:45	366	28.01.2015
88	176	conventional 2	F	13:15	366	28.01.2015
73	145	control	F	13:22	366	28.01.2015
73	146	control	F	13:34	366	28.01.2015
32	64	conventional 2	M	8:05	368	29.01.2015
32	63	conventional 2	M	8:25	368	29.01.2015
5	9	33% GMO	M	09:02	368	29.01.2015
5	10	33% GMO	M	09:10	368	29.01.2015
23	45	control	M	9:22	368	29.01.2015
23	46	control	M	9:38	368	29.01.2015
17	33	11% GMO	M	10:15	368	29.01.2015
17	34	11% GMO	M	10:21	368	29.01.2015
35	69	conventional 2	M	12:25	368	29.01.2015
35	70	conventional 2	M	12:31	368	29.01.2015
3	6	33% GMO	M	12:50	368	29.01.2015
3	5	33% GMO	M	13:01	368	29.01.2015

Sampling order

18	35	11% GMO	M	13:19	368	29.01.2015
18	36	11% GMO	M	13:28	368	29.01.2015
30	59	control	M	13:54	368	29.01.2015
30	60	control	M	14:10	368	29.01.2015
62	123	11% GMO	F	08:20	368	30.01.2015
74	148	control	F	8:43	368	30.01.2015
62	124	11% GMO	F	08:50	368	30.01.2015
74	147	control	F	9:05	368	30.01.2015
52	103	33% GMO	F	09:35	368	30.01.2015
52	104	33% GMO	F	09:47	368	30.01.2015
82	163	conventional 2	F	9:54	368	30.01.2015
82	164	conventional 2	F	10:05	368	30.01.2015
53	105	33% GMO	F	12:15	368	30.01.2015
53	106	33% GMO	F	12:25	368	30.01.2015
71	141	control	F	12:37	368	30.01.2015
71	142	control	F	12:50	368	30.01.2015
89	177	conventional 2	F	13:00	368	30.01.2015
89	178	conventional 2	F	13:15	368	30.01.2015
70	139	11% GMO	F	13:54	368	30.01.2015
70	140	11% GMO	F	14:09	368	30.01.2015
29	57	control	M	14:25	369	30.01.2015
29	58	control	M	14:40	369	30.01.2015
1	1	33% GMO	M	15:00	369	30.01.2015
1	2	33% GMO	M	15:28	369	30.01.2015
6	11	33% GMO	M	09:00	370	31.01.2015
6	12	33% GMO	M	09:14	370	31.01.2015
37	73	conventional 2	M	9:21	370	31.01.2015
37	74	conventional 2	M	9:34	370	31.01.2015
19	37	11% GMO	M	09:43	370	31.01.2015
19	38	11% GMO	M	09:45	370	31.01.2015
24	47	control	M	10:04	370	31.01.2015
24	48	control	M	10:19	370	31.01.2015
20	39	11% GMO	M	11:38	370	31.01.2015
20	40	11% GMO	M	11:48	370	31.01.2015
33	65	conventional 2	M	12:07	370	31.01.2015
33	66	conventional 2	M	12:13	370	31.01.2015
68	135	11% GMO	F	08:09	371	02.02.2015
68	136	11% GMO	F	08:20	371	02.02.2015
85	169	conventional 2	F	8:32	371	02.02.2015
85	170	conventional 2	F	8:55	371	02.02.2015
80	160	control	F	09:05	371	02.02.2015
55	109	33% GMO	F	09:17	371	02.02.2015
55	110	33% GMO	F	09:28	371	02.02.2015
81	161	conventional 2	F	11:52	371	02.02.2015
81	162	conventional 2	F	12:00	371	02.02.2015
67	133	11% GMO	F	12:40	371	02.02.2015
67	134	11% GMO	F	12:50	371	02.02.2015
75	149	control	F	13:00	371	02.02.2015
75	150	control	F	13:15	371	02.02.2015
59	118	33% GMO	F	13:23	371	02.02.2015

59	117	33% GMO	F	13:36	371	02.02.2015
8	16	33% GMO	M	07:53	373	03.02.2015
13	25	11% GMO	M	08:34	373	03.02.2015
13	26	11% GMO	M	08:44	373	03.02.2015
36	71	conventional 2	M	8:54	373	03.02.2015
25	49	control	M	9:20	373	03.02.2015
36	72	conventional 2	M	9:27	373	03.02.2015
25	50	control	M	9:40	373	03.02.2015
7	13	33% GMO	M	12:29	373	03.02.2015
7	14	33% GMO	M	12:37	373	03.02.2015
11	21	11% GMO	M	12:49	373	03.02.2015
11	22	11% GMO	M	12:58	373	03.02.2015
22	43	control	M	13:34	373	03.02.2015
22	44	control	M	13:50	373	03.02.2015
34	67	conventional 2	M	13:58	373	03.02.2015
34	68	conventional 2	M	14:09	373	03.02.2015
51	101	33% GMO	F	08:22	373	04.02.2015
51	102	33% GMO	F	08:37	373	04.02.2015
79	157	control	F	8:47	373	04.02.2015
79	158	control	F	8:58	373	04.02.2015
63	125	11% GMO	F	09:07	373	04.02.2015
63	126	11% GMO	F	09:17	373	04.02.2015
84	167	conventional 2	F	9:25	373	04.02.2015
84	168	conventional 2	F	9:35	373	04.02.2015
90	179	conventional 2	F	11:58	373	04.02.2015
90	180	conventional 2	F	12:10	373	04.02.2015
58	115	33% GMO	F	12:44	373	04.02.2015
58	116	33% GMO	F	12:48	373	04.02.2015
77	153	control	F	13:05	373	04.02.2015
77	154	control	F	13:16	373	04.02.2015
61	121	11% GMO	F	13:24	373	04.02.2015
61	122	11% GMO	F	13:37	373	04.02.2015
39	77	conventional 2	M	8:00	375	05.02.2015
39	78	conventional 2	M	8:10	375	05.02.2015
26	51	control	M	8:44	375	05.02.2015
26	52	control	M	8:55	375	05.02.2015
12	23	11% GMO	M	09:06	375	05.02.2015
12	24	11% GMO	M	09:15	375	05.02.2015
9	17	33% GMO	M	09:26	375	05.02.2015
9	18	33% GMO	M	09:40	375	05.02.2015
2	3	33% GMO	M	11:53	375	05.02.2015
2	4	33% GMO	M	12:05	375	05.02.2015
15	29	11% GMO	M	12:54	375	05.02.2015
15	30	11% GMO	M	13:10	375	05.02.2015
38	75	conventional 2	M	13:19	375	05.02.2015
38	76	conventional 2	M	13:28	375	05.02.2015
27	53	control	M	13:37	375	05.02.2015
27	54	control	M	13:50	375	05.02.2015
72	143	control	F	8:00	375	06.02.2015
72	144	control	F	8:09	375	06.02.2015

66	132	11% GMO	F	08:14	375	06.02.2015
83	165	conventional 2	F	8:27	375	06.02.2015
83	166	conventional 2	F	8:34	375	06.02.2015
57	113	33% GMO	F	09:09	375	06.02.2015
57	114	33% GMO	F	09:22	375	06.02.2015
60	119	33% GMO	F	11:48	375	06.02.2015
60	120	33% GMO	F	12:00	375	06.02.2015
87	173	conventional 2	F	12:10	375	06.02.2015
87	174	conventional 2	F	12:20	375	06.02.2015
69	137	11% GMO	F	12:47	375	06.02.2015
69	138	11% GMO	F	12:55	375	06.02.2015
78	155	control	F	13:03	375	06.02.2015
78	156	control	F	13:17	375	06.02.2015
