# Supplementary material for: One-year oral toxicity study on a genetically modified maize MON810 variety in Wistar Han RCC rats (EU 7th Framework Programme project GRACE)
Source: Arch Toxicol. 2016 Jul 20;90(10):2531–62. doi: 10.1007/s00204-016-1798-4 (PMC5043003; doi:10.1007/s00204-016-1798-4)
Supplement: Supplementary file 6 — Supplementary material 6 (DOCX 21 kb) [file 204_2016_1798_MOESM6_ESM.docx]

| **Week number** | **Males** | | | | **Females** | | | |
| --- | --- | --- | --- | --- | --- | --- | --- | --- |
|  | **control** | **conventional 2** | **11% GMO** | **33% GMO** | **control** | **conventional 2** | **11% GMO** | **33% GMO** |
| 0  1  2  3  4  5  6  7  8  9  10  11  12  13  15  17  19  21  23  25  27  29  31  33  35  37  39  41  43  45  47  49  51  53  end | 146.66 ± 5.26  201.92 ± 7.45  241.06 ± 10.01  274.60 ± 12.06  304.78 ± 14.30  328.28 ± 17.38  348.39 ± 21.25  364.17 ± 22.56  374.98 ± 24.65  388.79 ± 25.98  400.97 ± 28.42  415.26 ± 30.06  428.02 ± 32.39  435.46 ± 33.12  439.39 ± 34.40  457.89 ± 36.54  470.01 ± 38.72  479.97 ± 38.90  489.29 ± 43.27  499.92 ± 45.19  508.43 ± 44.76  521.78 ± 45.38  532.81 ± 46.25  541.09 ± 47.89  545.13 ± 47.79  547.62 ± 48.73  552.72 ± 52.12  557.26 ± 51.61  560.82 ± 52.63  567.15 ± 54.85  573.38 ± 55.04  582.08 ± 57.17  590.42 ± 59.01  592.88 ± 58.86  594.24 ± 59.95 | 147.57 ± 7.07  202.15 ± 8.61  239.47 ± 10.46  271.31 ± 11.86  299.18 ± 12.81  324.55 ± 13.53  344.83 ± 16.37  359.93 ± 17.55  370.80 ± 18.81  384.24 ± 21.24  398.06 ± 22.66  410.95 ± 24.80  424.64 ± 26.81  432.04 ± 28.08  435.96 ± 30.23  455.78 ± 33.71  467.72 ± 34.96  479.14 ± 36.39  484.95 ± 39.83  497.58 ± 41.27  506.41 ± 46.87  520.33 ± 47.48  528.43 ± 49.14  536.53 ± 49.49  546.04 ± 50.49  549.52 ± 53.54  553.68 ± 52.15  557.78 ± 52.38  561.15 ± 53.53  567.43 ± 53.42  575.60 ± 55.85  584.35 ± 56.58  591.54 ± 57.63  593.43 ± 58.37  598.61 ± 59.13 | 147.93 ± 4.44  205.30 ± 4.63  243.91 ± 5.81  277.88 ± 8.92  306.77 ± 8.55  332.87 ± 9.38  353.86 ± 9.59  370.67 ± 10.91  383.27 ± 11.94  398.54 ± 12.93  410.84 ± 13.93  423.10 ± 14.31  434.91 ± 15.29  441.49 ± 16.60  444.75 ± 17.61  465.99 ± 19.24  475.49 ± 19.61  484.82 ± 22.22  492.20 ± 23.53  501.99 ± 22.83  513.02 ± 25.17  523.51 ± 25.48  531.04 ± 27.67  538.69 ± 29.22  547.05 ± 27.98  551.22 ± 28.76  554.21 ± 28.99  558.66 ± 30.42  559.26 ± 29.18  567.36 ± 29.68  574.12 ± 30.26  581.73 ± 30.35  591.22 ± 31.95  588.05 ± 34.88  592.09 ± 34.03 | 147.36 ± 4.86  201.07 ± 4.53  236.89 ± 8.81  268.89 ± 11.95  294.59 ± 15.48  316.37 ± 19.01  331.41 ± 21.98  345.29 ± 24.71  356.11 ± 26.53  369.20 ± 29.00  381.85 ± 30.40  392.63 ± 29.32  405.22 ± 31.86  411.25 ± 32.07  413.86 ± 33.44  432.28 ± 35.04  444.74 ± 38.66  451.87 ± 40.17  457.44 ± 41.81  468.02 ± 41.63  476.45 ± 42.71  487.83 ± 44.09  496.74 ± 46.08  501.12 ± 47.11  509.64 ± 47.84  512.11 ± 50.50  514.57 ± 51.51  518.36 ± 53.19  520.83 ± 54.09  527.70 ± 53.19  531.60 ± 54.47  539.32 ± 56.50  547.98 ± 57.14  547.45 ± 57.60  550.31 ± 57.70 | 129.97 ± 4.45  163.18 ± 6.30  183.54 ± 9.59  202.22 ± 11.31  213.50 ± 14.44  226.52 ± 13.82  235.72 ± 13.11  241.70 ± 14.63  246.40 ± 15.24  251.69 ± 16.17  257.40 ± 16.77  262.47 ± 19.39  267.14 ± 19.64  271.81 ± 19.27  272.32 ± 18.51  280.06 ± 19.39  290.71 ± 18.70  295.32 ± 20.71  302.31 ± 21.91  309.14 ± 21.49  310.19 ± 21.91  319.57 ± 24.55  326.77 ± 24.77  331.15 ± 27.16  334.91 ± 27.79  331.92 ± 28.90  334.55 ± 32.24  338.51 ± 29.38  344.36 ± 30.52  344.96 ± 32.33  350.68 ± 35.69  357.56 ± 35.67  363.35 ± 35.52  363.90 ± 39.77  367.53 ± 41.93 | 129.96 ± 4.42  163.15 ± 5.25  181.02 ± 5.71  200.26 ± 6.11  210.79 ± 8.07  223.89 ± 8.93  232.80 ± 9.78  239.21 ± 9.97  246.13 ± 10.78  249.87 ± 11.65  254.96 ± 11.79  259.46 ± 12.94  265.21 ± 14.51  267.62 ± 14.14  268.66 ± 14.46  276.55 ± 15.99  284.62 ± 16.59  290.05 ± 18.06  295.09 ± 17.33  300.95 ± 19.14  298.42 ± 20.61  306.70 ± 23.18  311.74 ± 23.14  316.99 ± 24.65  322.31 ± 25.91  324.09 ± 25.01  326.26 ± 28.96  331.30 ± 31.15  336.07 ± 32.74  340.58 ± 36.71  347.20 ± 39.66  352.44 ± 42.53  355.81 ± 45.59  353.76 ± 45.14  358.02 ± 50.79 | 129.96 ± 4.45  159.96 ± 5.37  177.14 ± 5.59  194.03 ± 8.86  206.16 ± 10.77  219.31 ± 11.62  228.86 ± 10.95  235.70 ± 11.13  240.43 ± 11.52  244.86 ± 12.20  249.43 ± 12.75  253.44 ± 12.03  259.26 ± 14.63  262.94 ± 14.78  264.24 ± 15.31  271.79 ± 16.22  279.19 ± 15.99  283.02 ± 17.78  287.88 ± 18.59  293.23 ± 20.62  295.72 ± 20.93  301.37 ± 21.68  303.73 ± 20.03  306.07 ± 21.75  311.72 ± 23.71  307.63 ± 23.07  312.66 ± 22.24  317.03 ± 26.88  322.41 ± 25.98  326.22 ± 27.28  327.05 ± 30.02  331.14 ± 30.97  337.26 ± 32.34  337.73 ± 32.82  339.69 ± 33.27 | 130.03 ± 4.78  159.55 ± 7.39  176.72 ± 7.31  194.63 ± 9.67  205.53 ± 9.05  217.98 ± 9.02  226.07 ± 11.40  232.38 ± 11.53  238.58 ± 11.45  243.97 ± 11.26  247.48 ± 11.71  250.62 ± 12.16  255.97 ± 11.45  259.17 ± 10.76  259.91 ± 12.52  269.07 ± 12.38  277.37 ± 12.33  281.97 ± 12.25  284.81 ± 12.77  290.97 ± 13.94  291.81 ± 15.42  301.63 ± 14.72  306.32 ± 16.93  308.17 ± 16.37  313.59 ± 16.11  315.39 ± 19.30  316.02 ± 18.76  323.19 ± 19.10  327.44 ± 19.59  330.72 ± 22.77  335.17 ± 22.79  340.00 ± 23.64  344.83 ± 24.34  343.01 ± 24.08  344.62 ± 25.12 |

**ESM-Table 5:** Mean ± standard deviation of male and female rat body weight expressed per animal and in grams
