# Supplementary material for: One-year oral toxicity study on a genetically modified maize MON810 variety in Wistar Han RCC rats (EU 7th Framework Programme project GRACE)
Source: Arch Toxicol. 2016 Jul 20;90(10):2531–62. doi: 10.1007/s00204-016-1798-4 (PMC5043003; doi:10.1007/s00204-016-1798-4)
Supplement: Supplementary file 7 — Supplementary material 7 (DOCX 20 kb) [file 204_2016_1798_MOESM7_ESM.docx]

| **Week number** | **Males** | | | | **Females** | | | |
| --- | --- | --- | --- | --- | --- | --- | --- | --- |
|  | **control** | **conventional 2** | **11% GMO** | **33% GMO** | **control** | **conventional 2** | **11% GMO** | **33% GMO** |
| 1  2  3  4  5  6  7  8  9  10  11  12  13  15  17  19  21  23  25  27  29  31  33  35  37  39  41  43  45  47  49  51  53 | 247.01 ± 12.04  270.31 ± 15.10  278.99 ± 21.11  291.40 ± 16.34  301.86 ± 18.85  288.62 ± 20.82  278.47 ± 18.33  277.98 ± 19.36  281.32 ± 18.65  277.79 ± 19.48  288.30 ± 20.33  290.30 ± 19.55  276.40 ± 28.87  570.85 ± 44.90  557.39 ± 38.67  546.32 ± 39.21  523.65 ± 36.56  543.57 ± 43.08  538.34 ± 44.80  538.26 ± 42.48  557.70 ± 39.64  555.07 ± 39.09  568.83 ± 40.98  582.04 ± 41.59  583.05 ± 47.08  600.66 ± 55.05  606.94 ± 46.12  627.65 ± 48.77  632.10 ± 47.46  623.25 ± 39.75  624.45 ± 54.85  599.23 ± 53.22  576.72 ± 38.04 | 247.52 ± 16.71  272.96 ± 16.27  286.27 ± 15.16  286.15 ± 11.50  298.40 ± 10.69  291.44 ± 13.15  282.14 ± 14.61  282.71 ± 16.18  281.40 ± 14.54  278.97 ± 15.39  285.63 ± 19.22  288.76 ± 20.26  272.00 ± 29.59  561.36 ± 28.82  544.23 ± 44.56  539.49 ± 38.41  507.29 ± 44.15  521.24 ± 47.10  525.18 ± 51.73  526.81 ± 53.57  545.29 ± 43.83  538.88 ± 46.83  555.14 ± 48.99  545.56 ± 45.26  568.50 ± 56.40  604.97 ± 52.06  587.98 ± 58.62  600.36 ± 56.77  617.02 ± 48.54  598.37 ± 59.51  599.50 ± 59.39  590.25 ± 75.48  568.12 ± 39.05 | 259.82 ± 5.89  276.16 ± 12.38  287.49 ± 16.47  292.50 ± 14.98  309.13 ± 12.04  296.85 ± 8.79  290.22 ± 13.28  290.08 ± 13.65  288.94 ± 14.81  291.29 ± 13.07  287.07 ± 31.05  292.78 ± 15.24  277.52 ± 22.35  568.67 ± 26.59  551.06 ± 29.15  549.66 ± 26.38  531.30 ± 24.52  541.87 ± 24.52  542.36 ± 32.71  532.43 ± 31.82  549.54 ± 26.80  548.89 ± 33.29  556.72 ± 23.75  569.11 ± 22.77  565.42 ± 28.31  586.81 ± 30.22  588.37 ± 28.53  603.22 ± 29.16  612.84 ± 29.77  588.94 ± 26.09  601.70 ± 28.83  560.82 ± 29.66  553.73 ± 43.38 | 248.04 ± 13.23  265.35 ± 19.12  280.22 ± 18.15  282.32 ± 20.62  293.30 ± 22.05  276.88 ± 19.52  265.86 ± 20.24  270.48 ± 20.70  270.38 ± 20.56  274.28 ± 21.51  278.02 ± 19.95  282.29 ± 22.52  268.66 ± 25.81  545.82 ± 47.22  536.19 ± 47.13  538.72 ± 55.46  497.15 ± 45.38  508.92 ± 46.64  515.73 ± 41.49  515.48 ± 50.85  536.93 ± 53.43  531.34 ± 47.54  526.73 ± 47.25  516.21 ± 52.25  531.40 ± 51.99  557.80 ± 51.45  547.68 ± 48.27  552.49 ± 52.60  568.51 ± 49.95  551.95 ± 51.11  554.28 ± 47.97  526.66 ± 44.12  527.79 ± 47.87 | 177.93 ± 8.35  200.45 ± 14.69  213.68 ± 17.80  219.75 ± 16.60  222.07 ± 9.72  217.41 ± 14.23  215.45 ± 12.74  209.79 ± 10.27  208.69 ± 12.49  206.96 ± 16.82  216.53 ± 21.67  206.85 ± 10.05  208.80 ± 14.67  405.07 ± 27.32  411.79 ± 21.26  423.07 ± 36.27  412.83 ± 26.72  412.46 ± 33.87  409.93 ± 31.07  384.32 ± 38.49  421.45 ± 34.96  421.06 ± 25.84  418.59 ± 38.50  417.62 ± 34.36  430.95 ± 38.02  464.50 ± 35.31  466.03 ± 32.05  472.27 ± 43.68  471.16 ± 39.17  462.69 ± 40.30  477.39 ± 30.04  461.98 ± 50.67  404.44 ± 36.04 | 180.52 ± 10.36  195.13 ± 8.33  213.68 ± 11.59  219.32 ± 12.46  219.13 ± 10.80  215.86 ± 10.42  212.82 ± 8.75  214.11 ± 10.79  205.74 ± 9.48  206.40 ± 9.04  213.73 ± 9.91  211.29 ± 13.11  204.88 ± 14.48  403.11 ± 26.49  408.35 ± 22.78  414.26 ± 24.19  408.61 ± 17.91  409.00 ± 22.65  388.67 ± 28.49  375.29 ± 34.52  408.79 ± 30.02  407.20 ± 31.69  402.59 ± 27.89  420.35 ± 24.88  444.88 ± 28.78  462.63 ± 39.46  443.09 ± 25.12  463.70 ± 30.30  450.04 ± 34.04  460.06 ± 32.92  472.14 ± 28.77  459.16 ± 41.15  417.85 ± 32.41 | 184.73 ± 9.11  195.47 ± 10.53  215.30 ± 12.16  223.86 ± 15.92  188.23 ± 21.15  221.84 ± 12.88  263.42 ± 15.54  221.34 ± 16.01  215.90 ± 15.38  213.28 ± 13.73  218.53 ± 12.20  221.19 ± 24.05  220.81 ± 14.79  424.23 ± 27.36  428.19 ± 32.17  432.78 ± 29.46  425.02 ± 35.06  421.38 ± 33.94  424.31 ± 34.53  394.63 ± 31.41  416.80 ± 32.12  395.07 ± 27.56  402.72 ± 42.29  411.45 ± 81.77  438.35 ± 31.29  455.67 ± 35.81  476.01 ± 48.37  465.30 ± 37.22  458.45 ± 48.87  443.94 ± 38.94  467.85 ± 36.77  461.85 ± 36.73  411.77 ± 31.72 | 170.85 ± 20.36  191.35 ± 7.88  206.37 ± 12.91  211.80 ± 14.39  218.69 ± 14.94  215.59 ± 17.01  217.28 ± 13.33  210.48 ± 13.15  209.43 ± 14.24  204.93 ± 13.72  212.87 ± 16.59  210.03 ± 14.08  205.14 ± 17.11  403.69 ± 29.29  413.74 ± 29.62  415.45 ± 27.63  401.04 ± 23.22  392.52 ± 28.93  395.92 ± 26.32  387.23 ± 27.56  420.71 ± 26.42  394.32 ± 30.26  400.48 ± 24.37  405.43 ± 21.21  426.86 ± 23.53  447.81 ± 24.22  446.34 ± 20.34  447.46 ± 24.33  449.89 ± 23.44  441.72 ± 25.88  451.58 ± 27.17  434.65 ± 30.18  391.34 ± 33.35 |

**ESM-Table 6:** Mean ± standard deviation of male and female rat feed consumption expressed per cage, per week until week 13/per two weeks thereafter and in grams
