# Supplementary material for: One-year oral toxicity study on a genetically modified maize MON810 variety in Wistar Han RCC rats (EU 7th Framework Programme project GRACE)
Source: Arch Toxicol. 2016 Jul 20;90(10):2531–62. doi: 10.1007/s00204-016-1798-4 (PMC5043003; doi:10.1007/s00204-016-1798-4)
Supplement: Supplementary file 8 — Supplementary material 8 (DOCX 21 kb) [file 204_2016_1798_MOESM8_ESM.docx]

| **Week number** | **Males** | | | | **Females** | | | |
| --- | --- | --- | --- | --- | --- | --- | --- | --- |
|  | **control** | **conventional 2** | **11% GMO** | **33% GMO** | **control** | **conventional 2** | **11% GMO** | **33% GMO** |
| 1  2  3  4  5  6  7  8  9  10  11  12  13  15  17  19  21  23  25  27  29  31  33  35  37  39  41  43  45  47  49  51  53 | 22.35 ± 0.87  14.46 ± 0.63  12.00 ± 0.58  10.35 ± 0.71  7.75 ± 1.54  6.89 ± 2.31  5.65 ± 0.69  3.85 ± 0.98  4.89 ± 0.88  4.35 ± 0.87  4.94 ± 0.95  4.37 ± 0.97  2.62 ± 1.93  0.67 ± 0.57  3.31 ± 0.64  2.19 ± 0.82  1.92 ± 0.61  1.66 ± 1.03  1.96 ± 0.63  1.59 ± 0.75  2.39 ± 0.77  1.99 ± 0.57  1.44 ± 0.66  0.69 ± 0.47  0.42 ± 0.64  0.81 ± 0.62  0.75 ± 0.57  0.56 ± 0.37  0.98 ± 0.64  1.01 ± 0.65  1.36 ± 0.71  1.38 ± 0.68  0.44 ± 0.83 | 22.07 ± 0.88  13.66 ± 0.94  11.13 ± 1.04  9.75 ± 0.55  8.50 ± 1.13  6.93 ± 1.06  5.34 ± 0.66  3.82 ± 0.80  4.73 ± 1.63  4.93 ± 1.09  4.48 ± 1.00  4.71 ± 0.58  2.62 ± 1.71  0.68 ± 0.80  3.61 ± 0.86  2.21 ± 0.58  2.24 ± 0.78  1.06 ± 0.91  2.39 ± 0.43  1.58 ± 1.43  2.56 ± 0.57  1.49 ± 0.58  1.46 ± 0.41  1.74 ± 0.83  0.57 ± 0.67  0.71 ± 0.41  0.70 ± 0.41  0.55 ± 0.60  1.03 ± 0.58  1.34 ± 0.47  1.46 ± 0.42  1.20 ± 0.41  0.32 ± 0.69 | 22.07 ± 0.87  13.98 ± 0.96  11.78 ± 0.89  9.91 ± 1.07  8.46 ± 0.77  7.07 ± 0.62  5.77 ± 0.90  4.34 ± 0.71  5.26 ± 1.49  4.21 ± 1.16  4.34 ± 1.14  4.02 ± 0.79  2.31 ± 1.28  0.57 ± 0.29  3.83 ± 1.06  1.73 ± 0.40  1.73 ± 0.87  1.35 ± 0.76  1.82 ± 0.68  2.06 ± 1.03  1.90 ± 0.81  1.35 ± 0.65  1.36 ± 0.74  1.47 ± 0.68  0.74 ± 0.45  0.50 ± 0.75  0.74 ± 0.50  0.10 ± 0.88  1.31 ± 0.72  1.15 ± 0.55  1.26 ± 1.12  1.68 ± 1.37  -0.62 ± 1.02 | 21.62 ± 1.30  13.46 ± 1.00  11.39 ± 0.95  9.07 ± 1.10  7.38 ± 0.96  5.37 ± 1.20  5.17 ± 1.52  3.96 ± 0.89  4.80 ± 1.16  4.60 ± 0.67  3.90 ± 1.15  4.40 ± 0.95  2.17 ± 1.86  0.46 ± 0.37  3.43 ± 0.56  2.27 ± 0.56  1.42 ± 0.54  1.07 ± 0.63  2.05 ± 0.71  1.62 ± 0.96  2.12 ± 0.76  1.65 ± 0.66  1.65 ± 0.40  1.64 ± 0.64  0.46 ± 0.62  0.33 ± 0.64  0.76 ± 0.74  0.38 ± 0.70  1.27 ± 0.63  0.66 ± 0.48  1.35 ± 0.48  1.67 ± 0.70  -0.17 ± 0.60 | 18.61 ± 2.02  10.08 ± 1.47  8.70 ± 1.69  5.07 ± 1.41  5.86 ± 1.15  4.22 ± 1.32  2.75 ± 1.15  2.22 ± 0.99  2.52 ± 1.27  2.71 ± 1.42  2.26 ± 1.28  2.26 ± 0.74  2.24 ± 0.90  0.11 ± 1.12  1.87 ± 1.14  2.51 ± 0.92  1.08 ± 1.18  1.66 ± 0.92  1.61 ± 1.60  0.20 ± 1.58  2.20 ± 1.02  1.70 ± 0.86  0.99 ± 0.87  0.86 ± 1.35  -0.73 ± 0.81  0.53 ± 0.87  0.84 ± 1.42  1.23 ± 1.92  0.08 ± 1.32  1.18 ± 1.06  1.44 ± 0.52  1.38 ± 0.41  0.12 ± 1.74 | 18.40 ± 1.05  9.16 ± 1.04  8.97 ± 1.54  4.74 ± 1.50  5.97 ± 1.21  4.10 ± 1.15  2.99 ± 1.12  3.23 ± 0.73  1.80 ± 1.09  2.47 ± 0.98  2.08 ± 0.88  2.68 ± 1.27  1.13 ± 1.81  0.20 ± 1.34  1.92 ± 0.91  1.95 ± 1.08  1.32 ± 0.64  1.24 ± 0.71  1.50 ± 0.79  -0.75 ± 1.07  1.98 ± 0.86  1.23 ± 0.83  1.27 ± 0.85  1.25 ± 0.61  0.41 ± 1.23  0.40 ± 1.12  1.13 ± 0.84  1.01 ± 0.62  0.95 ± 1.29  1.41 ± 1.11  1.09 ± 1.05  0.72 ± 1.09  -0.55 ± 1.89 | 16.23 ± 1.34  8.79 ± 1.03  7.80 ± 1.80  5.40 ± 1.65  6.91 ± 1.26  4.31 ± 0.93  2.58 ± 1.33  2.14 ± 1.82  2.02 ± 1.17  2.14 ± 1.28  1.85 ± 1.15  2.51 ± 1.64  1.70 ± 1.01  0.30 ± 0.79  1.73 ± 0.58  1.72 ± 0.87  0.89 ± 1.23  1.11 ± 0.81  1.24 ± 1.13  0.59 ± 1.11  1.33 ± 0.82  0.60 ± 0.93  0.56 ± 1.06  1.53 ± 1.47  0.38 ± 0.60  0.96 ± 0.81  0.78 ± 1.83  0.99 ± 1.75  0.69 ± 1.26  0.20 ± 0.86  0.72 ± 0.82  1.30 ± 0.81  0.15 ± 2.00 | 17.25 ± 1.94  8.94 ± 1.36  8.64 ± 1.55  5.19 ± 1.35  5.66 ± 1.58  3.71 ± 1.51  2.89 ± 1.48  2.94 ± 1.25  2.57 ± 2.03  1.69 ± 1.94  1.43 ± 1.39  2.57 ± 0.99  1.52 ± 1.72  0.13 ± 1.44  2.21 ± 0.82  1.99 ± 0.69  1.15 ± 0.59  0.71 ± 0.92  1.54 ± 0.83  0.17 ± 1.27  2.36 ± 0.64  1.12 ± 1.42  0.45 ± 0.90  1.33 ± 0.89  0.39 ± 1.30  0.12 ± 0.59  1.60 ± 0.74  0.97 ± 1.29  0.71 ± 1.05  1.01 ± 0.49  1.06 ± 0.74  1.08 ± 0.85  -0.51 ± 1.26 |

**ESM-Table 7:** Mean ± standard deviation of male and female rat feed efficiency expressed per cage, per week until week 13/per two weeks thereafter and in percentage
