# Supplementary material for: One-year oral toxicity study on a genetically modified maize MON810 variety in Wistar Han RCC rats (EU 7th Framework Programme project GRACE)
Source: Arch Toxicol. 2016 Jul 20;90(10):2531–62. doi: 10.1007/s00204-016-1798-4 (PMC5043003; doi:10.1007/s00204-016-1798-4)
Supplement: Supplementary file 9 — Supplementary material 9 (DOCX 19 kb) [file 204_2016_1798_MOESM9_ESM.docx]

**ESM-Table 8:** Gross necropsy observations and corresponding histopathological findings in male and female Wistar Han RCC rats fed the 33% SY-NEPAL diet for 1 year

| **Male rats** | | |
| --- | --- | --- |
| **Animal No.** | **Gross necropsy observations** | **Histopathological findings** |
| 64 | white coloured formation on the surface of the urinary bladder | no histopathological alterations |
| 65 | bilateral hyperemic, dark coloured submandibular lymph node | no histopathological alterations |
| 68 | yellow coloured mass in the left epigastrium | lipoma |
| 72 | hematoma in the abdominal wall muscles | no histopathological alterations |
| 78 | size of the seminal vesicles and the coagulating glands decreased on the left side and increased on the right side, hyperemic jejunum | no histopathological alterations |
|  | | |
| **Female rats** | | |
| 162 | cystic formation in the left and right ovary, tissue mass in the caudal part of the mammary ridge | follicular cysts in the left and right ovary, mammary gland cystadenoma |
| 163 | hyperemic, dark coloured left submandibular lymph node | no histopathological alterations |
| 166 | cystic formation in the right ovary | follicular cysts in the right ovary |
| 169 | cystic formation in the left ovary | follicular cysts in the left ovary |
| 170 | mammary gland: strongly inflamed tissue, soft tissue mass protruding into the abdominal cavity; large blood clot above the hypophysis, enlarged hypophysis | mammary gland cystadenoma, pituitary haemangioma, focal proliferation of interstitial cells in the right adrenal gland |
| 171 | cystic formation in the left and right ovary | follicular cysts in the left and right ovary |
| 174 | cystic formation in the left ovary | follicular cysts and proliferation of stromal interstitial cells in the left ovary |
| 175 | cystic formation in the left and right ovary | left ovary: follicular cysts, proliferation and hypertrophy of stromal interstitial cells; right ovary: proliferation of stromal interstitial cells |
| 178 | cystic formation in the left and right ovary | follicular cysts and proliferation of stromal interstitial cells in the left and right ovary |
