# Supplementary material for: One-year oral toxicity study on a genetically modified maize MON810 variety in Wistar Han RCC rats (EU 7th Framework Programme project GRACE)
Source: Arch Toxicol. 2016 Jul 20;90(10):2531–62. doi: 10.1007/s00204-016-1798-4 (PMC5043003; doi:10.1007/s00204-016-1798-4)
Supplement: Supplementary file 10 — Supplementary material 10 (DOCX 19 kb) [file 204_2016_1798_MOESM10_ESM.docx]

**ESM-Table 9:** Gross necropsy observations and corresponding histopathological findings in female Wistar Han RCC rats fed the 11% GMO diet for 1 year

| **Animal No.** | **Gross necropsy observations** | **Histopathological findings** |
| --- | --- | --- |
| 123 | hyperemic, dark coloured submandibular lymph node | no histopathological alterations |
| 125 | dark red coloured surface at the bottom margin of the left kidney, red-brown coloured mass in the epigastrium,  hyperemic, dark coloured submandibular lymph node | lipoma; no further histopathological alterations |
| 126 | cystic formation in the left and right ovary | follicular cysts in the left and right ovary |
| 127 | cystic formation in the right ovary, thickened uterine wall | follicular cysts in the right ovary, endometrial stromal hyperplasia |
| 129 | light brown mass located in the right inguinal region, thickened uterine wall | lipoma, endometrial stromal hyperplasia, left ovary: atrophy, interstitial cell hyperplasia and hypertrophy, right ovary: follicular cysts |
| 130 | Iight hyperemic left and right ovary | follicular cysts in the left and right ovary |
| 131 | enlarged size of the left and right ovary, multiple nodules in the located on the surface of the liver, stomach, small and large intestine, mesenterial lymph nodes, spleen and urinary bladder | yolk sac carcinoma localized in the right ovary, multiple metastases in the abdominal cavity, endometrial stromal hyperplasia |
| 132 | cystic formation in the right ovary | follicular cysts and interstitial cell hyperplasia in the right ovary |
| 133 | cystic formation in the right ovary | follicular cysts and interstitial cell hyperplasia in the right ovary |
| 136 | wart in the lower part of the right ear | fibropapilloma |
| 137 | bilateral petechial hemorrhage (site: submandibular lymph node) | no histopathological alterations |
| 138 | cystic formation in the left ovary | follicular cysts in the left ovary |
| 139 | thickened uterine wall | endometrial stromal hyperplasia |
